# An Eight-Week, Web-Based Mindfulness Virtual Community Intervention for Students’ Mental Health: Randomized Controlled Trial

**DOI:** 10.2196/15520

**Published:** 2020-02-18

**Authors:** Farah Ahmad, Christo El Morr, Paul Ritvo, Nasih Othman, Rahim Moineddin, MVC Team

**Affiliations:** 1 School of Health Policy and Management York University Toronto, ON Canada; 2 Kinesiology & Health Science York University Toronto, ON Canada; 3 Dalla Lana School of Public Health University of Toronto Toronto, ON Canada; 4 See Authors' Contributions

**Keywords:** mindfulness, CBT, depression, anxiety, students, universities, randomized controlled trial, Canada

## Abstract

**Background:**

Innovative interventions are needed to address the increasing mental health needs of university students. Given the demonstrated anxiolytic and antidepressant benefits of mindfulness training, we developed an 8-week, Web-based Mindfulness Virtual Community (MVC) intervention informed by cognitive behavioral therapy (CBT) constructs.

**Objective:**

This study investigated the efficacy of the MVC intervention in reducing symptoms of depression, anxiety, and stress among undergraduate students in Toronto, Canada. The secondary outcomes included quality of life, life satisfaction, and mindfulness.

**Methods:**

The first 4 weeks of the full MVC intervention (F-MVC) comprised: (1) 12 video-based modules with psycho-education on students’ preidentified stressful topics and topically applied mindfulness practice; (2) anonymous peer-to-peer discussion forums; and (3) anonymous, group-based, professionally guided, 20-min live videoconferences. The second 4 weeks of F-MVC involved access only to video-based modules. The 8-week partial MVC (P-MVC) comprised 12 video-based modules. A randomized controlled trial was conducted with 4 parallel arms: F-MVC, P-MVC, waitlist control (WLC), and group-based face-to-face CBT; results for the latter group are presented elsewhere. Students recruited through multiple strategies consented and were randomized: WLC=40; F-MVC=40, P-MVC=39; all learned about allocation after consenting. The online surveys at baseline (T1), 4 weeks (T2), and 8 weeks (T3) included the Patient Health Questionnaire-9 item, Beck Anxiety Inventory, Perceived Stress Scale, Quality of Life Scale, Brief Multi-Dimensional Students Life Satisfaction Scale, and Five-Facet Mindfulness Questionnaire. Analyses employed generalized estimation equation methods with AR(1) covariance structures and were adjusted for possible confounders (gender, age, birth country, paid work, unpaid work, physical activities, self-rated health, and mental health counseling access).

**Results:**

Of the 113 students who provided T1 data, 28 were males and 85 were females with a mean age of 24.8 years. Participants in F-MVC (n=39), P-MVC (n=35), and WLC (n=39) groups were similar in sociodemographic characteristics at T1. At T3 follow-up, per adjusted comparisons, there were statistically significant reductions in depression scores for F-MVC (score change −4.03; *P*<.001) and P-MVC (score change −4.82; *P*<.001) when compared with WLC. At T3, there was a statistically significant reduction in anxiety scores only for P-MVC (score change −7.35; *P*=.01) when compared with WLC. There was a statistically significant reduction in scores for perceived stress for both F-MVC (score change −5.32; *P*<.001) and P-MVC (score change −5.61; *P*=.005) compared with WLC. There were statistically significant changes at T3 for quality of life and mindfulness for F-MVC and P-MVC vs WLC but not for life satisfaction.

**Conclusions:**

Internet-based mindfulness CBT–based interventions, such as F-MVC and P-MVC, can result in significant reductions in symptoms of depression, anxiety, and stress in a student population. Future research with a larger sample from multiple universities would more precisely test generalizability.

**Trial Registration:**

International Standard Randomized Controlled Trial Number ISRCTN92827275; https://www.isrctn.com/ISRCTN92827275

## Introduction

### Background

Mental health disorders, especially those involving depression, anxiety, and stress, are a rising problem among college students internationally. In the United States, analyses of college data show that mental health disorders are among the top five diagnostic categories seen at college health services and responsible for the highest number of visits per student (4.93) with depression and anxiety at the top [1]. Furthermore, multiple studies indicate an increasing prevalence of mental health disorders, especially depression and anxiety, in undergraduate students [1-8]. In Canada, a large study of nursing students indicated the prevalence of mild-to-severe depression, anxiety, and stress at 33%, 39%, and 38%, respectively [9]. Similar rates of mental health difficulties are reported among students from other countries [10-14]. The counseling centers in colleges and universities provide care to students in distress through various models such as clinical services, advising, awareness workshops, and training programs [15]. However, students often experience difficulties in accessing these services (eg, stigma and time concerns for in-person sessions along with financial cost for some services) [16,17], while counseling centers are overwhelmed due to limited resources. An analysis of Canadian colleges and universities revealed that enrollment in the province of Ontario increased by 27% between 2004 and 2012, but the budget for counseling centers increased by 5%, leading to just 1 campus-based counselor for 1300 to 4835 students [18]. Similarly, in the United States, a 2014 study indicated that the average ratio of counselors to students was 1 to 2081 [19]. New and accessible strategies are needed to address the students’ mental health and at an early stage. One such approach is mindfulness-based techniques.

Mindfulness is defined as “the awareness that emerges through paying attention on purpose, in the present, and nonjudgmentally to the unfolding of experience moment by moment” [20]. The techniques learned in mindfulness practices involve nonjudgmental attention directed to each present moment. Although mindfulness meditation has been practiced for centuries in Buddhist and other spiritual traditions, its application to psychological health in the West emerged in 1980s when Jon Kabat-Zinn examined its clinical use in treating chronic pain [21]. This technique known as mindfulness-based stress reduction has a core focus on “intensive [and repeated] training of mindfulness meditation to help individuals relate to their physical and psychological conditions in a more accepting and nonjudgmental ways” [22]. Further scholarly work, such as by Segal et al [23], combined the principles of mindfulness with cognitive behavioral therapy (CBT). This program called mindfulness-based cognitive therapy (MBCT) has been researched for treating mental health conditions, especially depression. In addition to the principles of mindfulness practice, MBCT “aims to change one’s *awareness of* and *relationship to* thoughts and emotions” to reduce the associations between negative automatic thinking and dysphoria [22]. Other psychotherapeutic techniques with mindfulness-orientation include dialectical behavior therapy and acceptance and commitment therapy, but the meditation practice is only one aspect of the full approach. Evidence shows that mindfulness-based interventions positively impact psychological [22,24] and physical health [25], with multiple meta-analyses demonstrating positive impacts in clinical and nonclinical populations [26-30]. Recent randomized controlled trials (RCTs) on mindfulness using face-to-face sessions, prescribed exploratory mental exercises, and video programs have reflected effectiveness in reducing symptoms for one or more of the three conditions of anxiety, stress, and depression [31-38]. In relation to student population, several recent reviews have indicated that in-person mindfulness-based interventions have a positive effect on students’ mood and their levels of stress, anxiety, and depression [39-42].

However, a handful of student studies exist on *Web-based* mindfulness-based programs despite its potential to complement overstretched traditional counseling services on campuses [43]. This emerging scholarly work with students has examined the impact of Web-based mindfulness on a variety of mental health–related issues and demonstrated improvements in outcomes such as mental health, well-being, mindfulness, stress and depression symptoms, life satisfaction, and social connectedness [43-50]. However, the effectiveness of a Web-based mindfulness intervention when combined with the constructs of CBT remains an area requiring more rigorous examination. This is a missed opportunity given that systematic reviews show that internet-based CBT is significantly effective compared with control groups in reducing anxiety, especially when supported by therapist’s email or phone contact [51], and in reducing depression symptoms [52]. There is the potential for substantial gains by combining these two techniques—mindfulness and CBT—through Web-based interventions for students who are also technologically fluent and capable; studies also indicate that students prefer to self-initiate help-seeking for Web-based services compared with in-person services [53]. There is also a need to better understand the optimal duration and delivery style of Web-based mindfulness-CBT interventions. Although durations of 6 to 8 weeks are more common, 2-week interventions [48] and a single Web session [47] have also been used. In terms of delivery, some of these studies supported the interventions with reminders, written feedback, and coaching, whereas others were passive. High attrition rate was a common problem in several student studies [44,45,48,50], although it was a significantly less prevalent problem in studies that used coaching, reminders, and feedback strategies [43,49]. Indeed, further scholarly work is needed to inform development of student-friendly and effective Web-based mindfulness-CBT programs. Thus, our team developed a Mindfulness Virtual Community (MVC) Web-based program (described below) after conducting eight focus groups with students and incorporating comprehensive review of pertinent literature [54-58].

### Study Objective

To examine the efficacy of an MVC program for mental health among undergraduate students in a Canadian university, we conducted a pilot RCT with 4 parallel arms: full MVC (F-MVC), partial MVC (P-MVC), waitlist control (WLC), and group-based face-to-face CBT mindfulness. As the main focus of the trial was to examine the MVC program, we report here the impact of F-MVC and P-MVC vs WLC; the results for face-to-face CBT mindfulness are presented elsewhere. The primary outcomes were symptoms of depression, anxiety, and stress, and secondary outcomes were quality of life, life satisfaction, and mindfulness. It was hypothesized that (1) symptom scores for depression, anxiety, and stress at 8 weeks (T3) will be significantly improved in the F-MVC group when compared with the WLC group and (2) scores for quality of life, life satisfaction, and mindfulness at T3 will be significantly better for the F-MVC intervention group than the WLC group. The P-MVC intervention was included to explore a significantly less expensive alternative to delivering beneficial effects and was hypothesized to have similar but lesser impact than the F-MVC intervention.

## Methods

### Ethics and Timeline

The Human Participant Research Committee at York University, Toronto, provided research ethics approval. We followed Consolidated Standards of Reporting Trials guidelines for nonpharmacological interventions and electronic health interventions [59,60]. The recruitment of eligible undergraduate students occurred during December 7, 2016 and January 10, 2017. These students started the parallel-arm RCT on January 16, 2017, with a baseline survey (T1) followed by exposure to 2 interventions, a 4-week online survey (T2), and an 8-week online survey (T3). The 8-week-long interventions of F-MVC and P-MVC started on January 22, 2017 and ended on March 16, 2017.

### The Mindfulness Virtual Community Program

**Figure 1:**
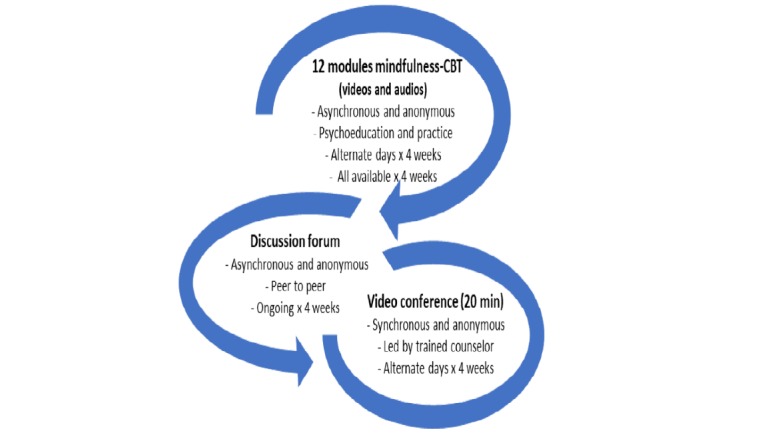
Mindfulness Virtual Community program informed by cognitive behavioral therapy constructs. CBT: cognitive behavioral therapy.

A total of three components of the Web-based MVC program (Figure 1) were (1) youth-specific mental health education and mindfulness-practice modules, delivered via video recordings for participants to watch and listen to on personal computers, phones, and tablets at convenient times; (2) anonymous, asynchronous peer-to-peer discussion boards pertaining to mental health and mindfulness practice; and (3) anonymous, 20-min live videoconferences (group-based) on module topics guided by a mental health professional.

Each of the 12 modules consists of one educational content and one mindfulness-practice content video, recorded in male and female voices with low volume background music, and offered in high- and low-resolution videos (a total of 8 videos per module). The modules’ topics were informed by our findings from the focus groups with students [54,55]. The module scripts and audio recordings were created by one of the investigators with extensive clinical experience (PR) and drew from combined mindfulness and CBT principles. The choice of moving and still images used in the creation of the videos involved collaborative work (PR, CE, and FA). Table 1 lists the topics of 12 modules and video duration (average of male and female voice), and Textbox 1 provides examples of the module content.

**Table 1:** Topics and duration of modules.

Topics	Video duration (minutes:seconds)	
	Education	Mindfulness practice
Overcoming stress, anxiety, and depression	7:09	9:00
Mindfulness and being a student	5:18	9:14
Mindfulness for better sleep	4:40	8:13
Thriving in a fast-changing world	7:23	8:23
Healthy intimacy	7:32	9:33
Destigmatization	6:13	9:12
No more procrastination	3:42	10:48
Pain reduction and mindfulness	3:48	9:48
Healthy body image	5:44	9:54
Healthier eating	10:10	9:26
Overcoming trauma	6:01	9:43
Relationships with family and friends	7:49	8:09

**Examples of module content.**

**Module 1: Overcoming stress, anxiety, and depression**

- *Education video*: The initial narration focuses on sources of stress (eg, continuous online access, information overload, and worries about the past and future distracting from focusing on the present). Video clips of human faces and activities depict mixtures of stress, relaxation, and joy. The middle section introduces mindfulness training with breath awareness as one approach to developing a present moment orientation that replaces less desirable coping attempts that involve purposeful self-distraction (eg, screen time). The video clips reflect people stressed (eg, at work, unable to sleep) and other people in a more relaxed states (eg, by a pool or on a beach or walking). The last narrative section encourages releases of accumulated stress and tension via mindfulness practice. Self-selected practice times are emphasized that may include a session at the end of day, reflected visually by clips of sunsets and people commuting home or just reading in a relaxed way within home environments.
- *Mindfulness practice*: Video clips of the flow of a natural river accompanies narration focused on instructions to find a comfortable position and focus on breathing sensations, with attention particularly directed to exhaling breaths; further instructions focus on acknowledging wandering thoughts and, after noticing them, returning attention to breathing; final suggestions (on this 9-min segment) are to accept stressful thoughts and then let them go, releasing the associated tensions. Altogether the session is characterized as a simplicity break where the focus on natural breathing rhythms assists one in attending to present moment experiences; narration concludes with the suggestion that the listener can continue to practice for periods of time that seems personally appropriate.

**Module 2: Mindfulness and being a student**

- *Education video*: The initial section focuses on student challenges (eg, memorization and exam taking) with video clips of students studying for and completing exams; the second section explains how mindfulness stimulates parasympathetic dominance and a psychological and physical calm, referring to experimental evidence on related benefits for learning, memory, and motivation. Video clips show people playing basketball and music (in confident ways), whereas the narrative section encourages mindfulness as a way of increasing confidence in personal skill development.
- *Mindfulness practice*: Images of very tall trees with light filtering through them accompany narration instruction on mindfulness practice with several points referring to the typical student experiences highlighted in the previous education video.

**Figure 2:**
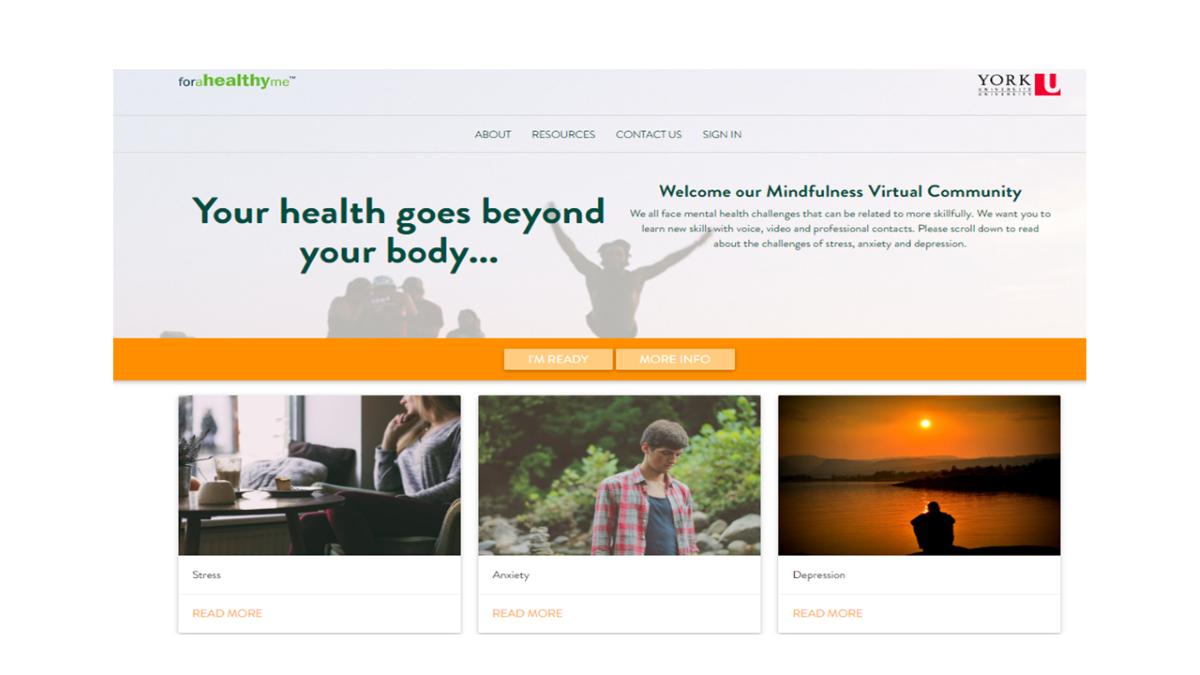
Screenshot of the Mindfulness Virtual Community platform.

The Web platform for the intervention had separate logins for the student participant and the videoconference moderator (Figure 2). This was developed in partnership with the industry partner, ForaHealthyme Inc. The F-MVC student version provided access to the video-based modules, text-based peer-to-peer discussion forum, a calendar to book an upcoming live videoconference, a video room (camera being off as default) with ability to privately text the moderator, and a resource page with contact information on various social and health services.

The moderator version offered access to the student version along with additional features such as populating the calendar with dates and times for the live videoconferences, starting the videoconference with camera turned on for the moderator by default, and responding privately to incoming text messages. The moderator (independent counselor with a master’s degree in psychology and training in mindfulness) had weekly discussions with the team clinician (PR) to optimize engagement during videoconferences. Once the intervention was deployed, the content of modules and platform structure remained unchanged.

### Study Arms

The Web-based F-MVC intervention was 8 weeks long. In the first 4 weeks, it offered 12 video-based modules, peer-to-peer discussion forums, and brief guided videoconferences; and in the second 4 weeks, it offered continued access to the video-based modules. The release of new modules in the first 4 weeks was scheduled for Sundays, Tuesdays, and Thursdays, and the video chat sessions were offered on Mondays, Wednesdays, and Fridays, with 20-min evening sessions at 9:00 PM, 9:30 PM, and 10:00 PM. Once a module was released, it remained accessible to students for the remainder of the intervention period. The Web-based P-MVC intervention was 8 weeks long and included all the video material of the F-MVC intervention following a similar release schedule, but it did not offer any videoconferences or discussion forums. The students in both groups received email reminders from the project staff before the release of each new module, whereas the reminders for conference chat were sent only to the F-MVC group. The partial and full intervention participants were instructed to use the platform ad libitum. The WLC group continued as *usual care* during the 8-week period without access to additional resources, and after completing the T3 survey, they received access to the Web-based 12 video-based modules and information on ongoing face-to-face mindfulness groups at the university to join if interested.

### Recruitment and Randomization

Student eligibility criteria were a minimal age of 18 years, English language fluency, self-reported high level of confidence to complete the study, and current undergraduate student status. Their ability to use a computer and smartphone and internet literacy were assumed to be de facto skills. The study was advertised as “Mindfulness Approaches to Wellbeing on Campus” and used multiple recruitment strategies including study posters, class visits on permission of course directors, and email invitations via listservs of student associations in the Faculty of Health and Faculty of Liberal Arts. Interested students contacted the research staff via email or phone and were further screened for substance abuse and indications of psychoses (ie, hallucinations). If either of these two conditions “interfered in routine life within last month,” they were excluded and provided a list of mental health resources for access. Eligible and willing students received detailed information in-person about the study and provided informed written consent. Participants were able to select an honorarium of Can $50 or 2% in course grade (for professors who gave this permission) or three credits (equivalent to 2% course grade) in the Undergraduate Research Participation Pool of the Department of Psychology. Each participant also received a resource list that included information about health and social services on campus and in the community (eg, 24×7 “Good To Talk” helpline for postsecondary students in Ontario). Although our study participants largely comprised healthy volunteers, our protocol included a safety mechanism whereby participants were asked verbally and in the consent form to contact the research staff if they felt distress during the trial period so that “limited counselling with a clinical psychologist could be arranged, if needed”; the collaborating psychologist was at arms’ length from the trial. No instance of such request arose during the reported study.

Participating students were randomized to either the F-MVC intervention, P-MVC intervention, WLC, or a face-to-face CBT mindfulness group using 1:1:1:1 block randomization. We report here the impact of F-MVC and P-MVC vs WLC, whereas the results for face-to-face CBT mindfulness are being presented elsewhere (manuscript under review). The randomization allocation sequence was computer-generated by an off-site team member who concealed it in sequentially numbered, opaque envelopes [61]. These envelopes were opened only after a written consent, keeping participants and research assistants blind to allocation. Each participant received a unique ID number. Those in the F-MVC and P-MVC groups also received a temporary password to access the Web-based intervention; they changed the password after first login while IDs remained the same to eliminate the possibility of creating multiple accounts or identities.

### Main Outcomes and Measurement

Participants in all groups completed online surveys at T1, T2, and T3. The primary outcomes were depression, anxiety, and stress symptoms. For the measurement of depression symptoms, we used the 9-item Patient Health Questionnaire (PHQ-9) [62]; each item is rated on a scale of 0 to 3, and the total score range is 0 to 27 (score 0-9 indicates no/subclinical level of depression, 10-14 moderate, 15-19 moderately severe, and ≥20 severe). The symptoms of anxiety were measured by using the 21-item Beck Anxiety Inventory (BAI) [63]; each item is rated on a scale of 0 to 3, and the total score range is 0 to 63 (score 0-21 indicates no/low level of anxiety, 22-35 moderate, and ≥36 severe). For the measurement of stress, we used the 10-item Perceived Stress Scale (PSS) [64]; each item is rated on a scale of 0 to 4, and the total score range is 0 to 40 (score 0-13 indicates mild level of stress, 14-26 moderate, and 27-40 high). The secondary outcomes were quality of life, life satisfaction, and mindfulness. We used the 16-item Quality of Life Scale (QOLS) [65], which has a total score range of 16 to 112, and each item is rated on a scale of 1 to 7. The student life satisfaction was measured by using the 6-item Brief Multidimensional Students’ Life Satisfaction Scale-Peabody Treatment Progress Battery (BMSLSS-PTPB) [66]; each item is rated on a scale of 1 to 5, and item scores are averaged together to give a total score that ranges from 1 to 5. The level of mindfulness was measured by the 24-item Five-Facet Mindfulness Questionnaire-Short Form (FFMQ-SF) [67]; each item is rated on a scale of 1 to 5, and the total score range is 24 to 120. The subscales in the FFMQ-SF are nonreactivity to inner experience (5 items), observing (4 items), acting with awareness (5 items), describing (5 items), and nonjudging of inner experience (5 items). We assessed each of the scale for internal consistency at the T1, T2, and T3 datasets, and Cronbach alpha ranged from .82 to .94: PHQ-9 .87, .89, and .86; BAI .93, .93, and .94; PSS .90, .89, and .90; QOLS .87, .91, and .92; BMSLSS-PTPB .82, .85, and .85; and FFMQ-SF .86, .87, and .89.

Participants also completed a sociodemographic questionnaire at the T1 survey that inquired about age, gender, birth country, years lived in Canada, first language, ethnic heritage, intimate relationship status, self-rated health (from poor to excellent), access to private mental health counseling, paid and unpaid work, and average minutes spent per week on rigorous physical activities. The T3 survey also asked all participants to report their self-perceived change in the academic performance (worse, same, or better) and in class attendance/absenteeism (more frequent, about same, or less frequent) since the start of this study. The T3 survey for the F-MVC and/or P-MVC groups also included questions on module use (number of videos watched in full, average frequency of watching each video), exchanges during discussion forums (for appropriateness, supportiveness, and informativeness), and videoconferences (for ease in access, convenience, help in understanding personal mindfulness practice and mental well-being, and help via the direct messaging feature). Participants answered using a scale of 1 to 5 (1=strongly disagree, 2=disagree, 3=neutral, 4=agree, and 5=strongly agree) for questions on the discussion forum and videoconferences.

### Sample Size and Analysis

The outcomes of the study are continuous and measured at T1, T2, and T3. We calculated the sample size assuming the intraclass correlation coefficient to be 0.6 and standardized effect size to be 0.5 or larger. Following Hedeker et al’s [68] results for sample size calculation for longitudinal study, a sample size of 47 students in each arm provides 80% power to detect significance of standardized changes of size 0.5 or larger with 5% type I error.

The trial data were first analyzed using descriptive statistics (means, frequencies, and proportions) to describe the sample characteristics for the control, partial intervention, and the full intervention groups at T1. The mean scores for each of the 6 scales (ie, primary and secondary outcomes) were calculated for the T1, T2, and T3 for the three groups. Effect size was calculated using Cohen *d*, by subtracting the mean of the treatment group from the mean of the control group and by dividing the mean difference with the pooled SD.

The approach to the outcome analysis was Intention-to-Treat. First, we analyzed the data without any imputation for missing values and then repeated the analysis with an imputation of missing values using a last observation carried forward (LOCF) method. The results were similar for the complete-case analysis and analysis with LOCF; both are reported (see Multimedia Appendices 1 and 2). The attrition rates across the three groups were low and similar between T1 and T3 (2 for F-MVC, 1 for P-MVC, and 1 for WLC). To compare score changes over time for the outcomes, linear regression analysis was done. The generalized estimation equation (GEE) with AR(1) covariance structure was used to adjust for repeated measures. The result of GEE analysis has the interpretation of population average. The mean score differences were calculated between groups and adjusted for potential confounding variables (ie, gender, age, country of birth, paid work, unpaid work, vigorous physical activities, self-rated health, and access to mental health private counseling via insurance). This choice of confounding variables was based on existing knowledge and theory. Scales were calculated as follows: if the number of missing items was more than half of the number of items of a scale, the scale was considered missing; otherwise the missing items were imputed in accord with the average of the nonmissing items of the scale. To account for multiple comparisons, due to the multiple outcomes analyzed, we considered *P*<.02 as statistically significant. The data analysis was conducted by biostatistician on our team (RM) who was not involved in the content development of the intervention and its deployment. The statistical software SAS 9.4 was used for statistical analyses.

## Results

### Participants

**Figure 3:**
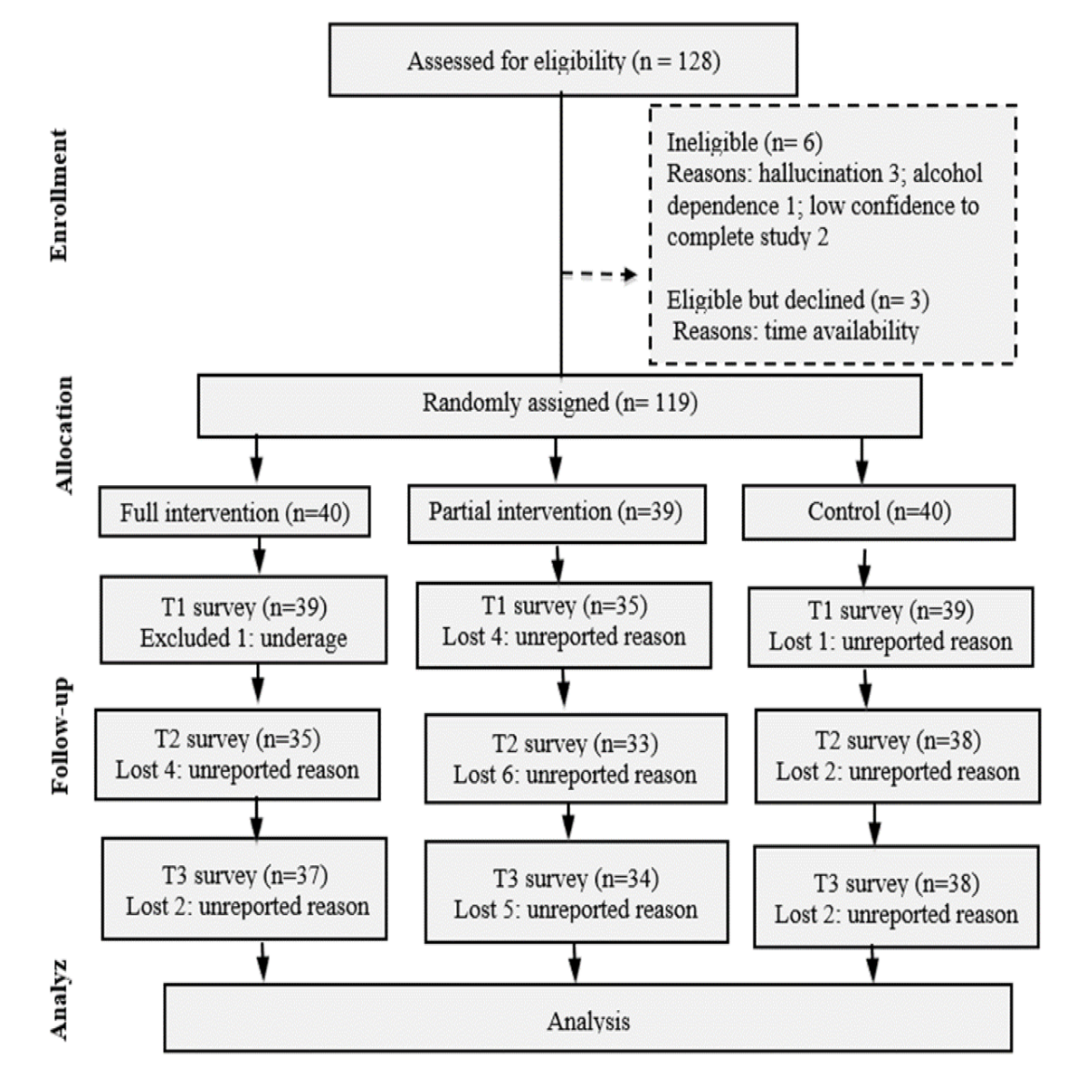
Study flow diagram. T1: baseline; T2: 4 weeks; T3: 8 weeks.

A total of 119 undergraduate students were randomized to the WLC, F-MVC, and P-MVC groups; 1 participant, following consent, was found to be underage and was therefore excluded, and 5 additional participants were nonrespondents to the T1 survey. Out of the 113 students who completed the T1 survey, a few were lost as nonrespondents at the follow-up; altogether the attrition was relatively low across all three groups (F-MVC: 2/39, P-MVC: 1/35, and WLC: 1/39; Figure 3).

Overall, there were 24.8% (28/113) males and 75.3% (85/113) females. The majority of participants, 59.3% (67/113), were born in Canada, and 64.6% (73/113) reported English as their first language, whereas 37.2% (42/113) self-identified as white. These and other characteristics seemed to be similarly distributed between the control and intervention groups (Table 2).

**Table 2:** Participant characteristics.

Characteristics		All (N=113)	Control (n=39)	Full intervention (n=39)	Partial intervention (n=35)
Age (years), mean (SD)		24.8 (6.5)	25.4 (7.3)	24.9 (6.4)	24.1 (5.7)
**Gender, n (%)**					
	Male	28 (24.8)	8 (21)	10 (26)	10 (29)
	Female	85 (75.2)	31 (80)	29 (74)	25 (71)
**Country of birth, n (%)**					
	Canada	67 (59.3)	22 (56)	26 (67)	19 (54)
	Other	46 (40.7)	17 (44)	13 (33)	16 (46)
Years in Canada, mean (SD)		4.5 (7.4)	5.6 (8.4)	2.2 (4.7)	5.7 (8.1)
**First language, n (%)**					
	English	73 (64.6)	23 (59)	26 (67)	11 (31)
	Other	40 (35.4)	16 (41)	13 (33)	24 (69)
**Relationship status, n (%)**					
	Single, no relationship	68 (60.2)	17 (44)	32 (82)	32 (54)
	Single in relationship	37 (32.7)	17 (44)	6 (15)	14 (40)
	Married/common law	8 (7.1)	5 (13)	1 (3)	2 (6)
**Ethnicity, n (%)**					
	White	42 (37.2)	17 (44)	13 (33)	12 (34)
	Black	11 (9.7)	4 (10)	4 (10)	3 (9)
	South Asian	22 (19.5)	7 (18)	5 (13)	10 (29)
	Chinese	12 (10.6)	1 (2)	7 (18)	4 (11)
	Other	26 (23.0)	10 (26)	10 (26)	6 (17)
**Self-rated health, n (%)**					
	Poor/fair	29 (25.9)	11 (29)	6 (15)	12 (34)
	Good	45 (40.2)	11 (29)	19 (49)	15 (43)
	Very good/excellent	38 (33.9)	16 (42)	14 (36)	8 (23)
**Access to private mental health, n (%)**					
	Yes	49 (43.8)	13 (34)	19 (49)	17 (49)
	No	63 (56.2)	25 (66)	20 (51)	18 (51)
**Weekly hours, mean (SD)**					
	Paid work	7.6 (9.9)	10.1 (11.1)	7.9 (10.5)	4.6 (7.0)
	Unpaid work	3.7 (4.9)	3.4 (4.4)	4.4 (6.0)	3.2 (4.3)
Weekly vigorous physical activities in minutes, mean (SD)		30.2 (57.8)	25.3 (52.4)	16.4 (26.3)	50.7 (80.4)

### Depression, Anxiety, and Stress Symptoms

Table 3 provides the proportion of participants at T1 and T3 for various levels of symptoms for depression, anxiety, and stress; 1 participant did not complete the mental health scales. In the total sample, 36.6% (41/112) had PHQ-9 scores ≥10, and 20.5% (23/112) had a BAI score ≥22, indicating probable clinical depression and anxiety. In the F-MVC group, 28% (11/39) and 19% (7/37) had PHQ-9 scores ≥10 at T1 and T3, respectively, and 23% (9/39) and 14% (5/37) had BAI scores ≥22 at T1 and T3, respectively. In the P-MVC group, the proportions for T1 and T3 were 40% (14/35) and 24% (8/33) for PHQ-9 ≥10, and 20% (7/35) and 18% (6/33) for BAI ≥22, respectively.

**Figure 4:**
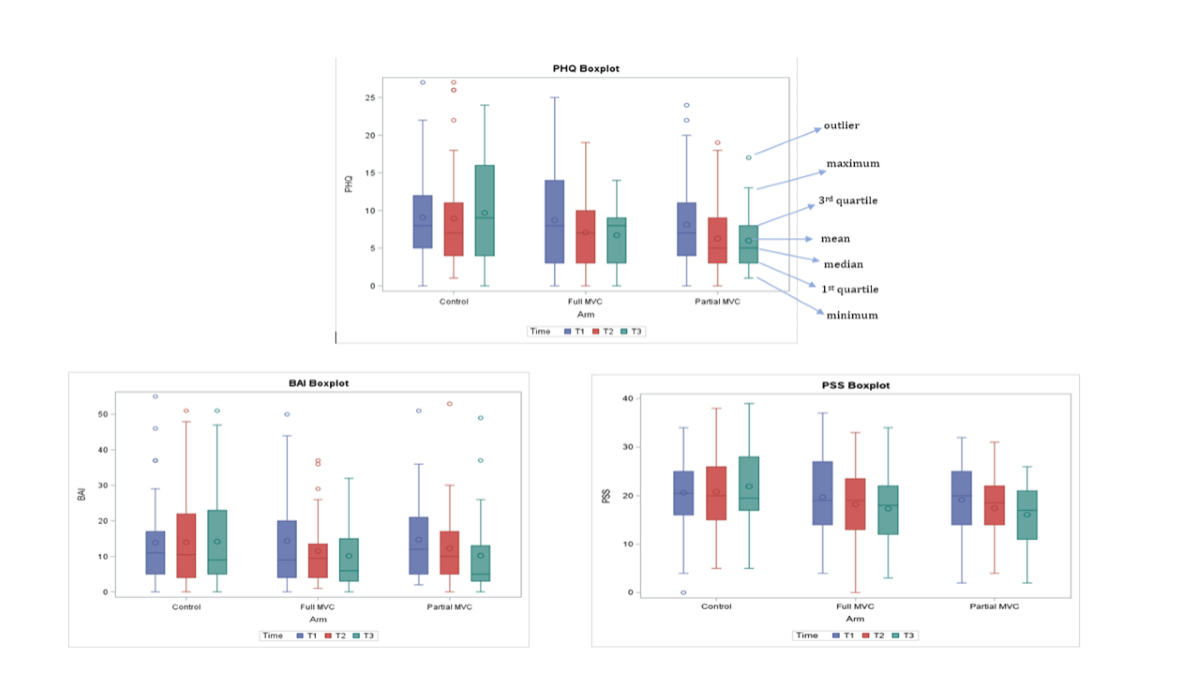
Box plots for the mean scores of depression, anxiety, and stress scales. BAI: Beck Anxiety Inventory; MVC: Mindfulness Virtual Community; PHQ: Patient Health Questionnaire; PSS: Perceived Stress Scale; T1: baseline; T2: 4 weeks; T3: 8 weeks.

The T1, T2, and T3 means and SDs are presented in Tables 4 and 5 for the 6 scales used in the study. These tables also provide the Cohen *d* effect size for mean difference in the F-MVC group compared with the control group and the mean difference in the P-MVC group compared with the control group at both T2 and T3. Figure 4 presents the mean scores for primary outcomes as box plots.

**Table 3:** Symptom levels for depression, anxiety, and stress scales.

Characteristics		Control (n=38)		Full intervention (n=39)		Partial intervention (n=35)	
		n (%)	95% CI	n (%)	95% CI	n (%)	95% CI
**Patient Health Questionnaire** **9-item**							
	T1^a^ score 0-9	22 (58)	40.1-73.7	28 (72)	55.1-85.0	21 (60)	42.1-76.1
	T1 score ≥10	16 (42)	26.3-59.2	11 (28)	15.0-44.9	14 (40)	23.9-57.9
	T3^b^ score 0-9	21 (55)	38.3-71.4	30 (81)	64.8-92.0	25 (76)	57.7-88.9
	T3 score ≥10	17 (45)	28.6-61.7	7 (19)	8.0-35.2	8 (24)	11.1-42.3
**Beck Anxiety Inventory** **21-item**							
	T1 score 0-21	31 (82)	69.3-93.9	30 (77)	63.7-90.1	28 (80)	66.7-93.3
	T1 score ≥22	7 (18)	7.7-34.3	9 (23)	11.1-39.3	7 (20)	8.4-36.9
	T3 score 0-21	26 (68)	53.6-83.2	32 (87)	75.5-97.5	27 (82)	68.7-95
	T3 score ≥22	12 (32)	17.5-48.7	5 (14)	4.5-28.8	6 (18)	5.0-31.3
**Perceived Stress Scale** **10-item**							
	T1 score 0-13	4 (11)	0.8-20.3	9 (23)	9.9-36.3	8 (23)	8.9-36.8
	T1 score ≥14	34 (90)	75.2-97.1	30 (77)	60.7-88.9	27 (77)	59.9-89.6
	T3 score 0-13	6 (16)	4.2-27.4	13 (35)	19.8-50.5	11 (33)	17.2-49.4
	T3 score ≥14	32 (84)	68.7-94.0	24 (65)	49.5-80.2	22 (67)	48.2-82.0

^a^T1: baseline.

^b^T3: 8 weeks.

**Table 4:** Mean (SD) and effect size for depression, anxiety, and stress scales.

Time of measurement		Control (n=38)	Full intervention		Partial intervention	
		Mean (SD)	Mean (SD)	Effect size	Mean (SD)	Effect size
**Patient Health Questionnaire** **9-item**						
	T1^a^	9.1 (6.2)	8.1 (6)^b^	N/A^c^	8.7 (6.3)^d^	N/A
	T2^e^	8.9 (6.9)	6.3 (4.8)^f^	−0.43	7.1 (4.8)^g^	−0.30
	T3^h^	9.7 (6.9)	6 (3.9)^i^	−0.66	6.7 (3.9)^j^	−0.53
**Beck Anxiety Inventory** **21-item**						
	T1	13.9 (12.9)	14.7 (11.5)^b^	N/A	14.4 (12.8)^d^	N/A
	T2	14 (13.2)	12.2 (10.6)^f^	−0.15	11.5 (9.6)^g^	−0.21
	T3	14.2 (12.6)	10.2 (11.1)^i^	−0.34	10.2 (9.6)^j^	−0.35
**Perceived Stress Scale** **10-item**						
	T1	20.6 (7.8)	19.2 (7.5)^b^	N/A	19.7 (7.8)^d^	N/A
	T2	20.8 (7.9)	17.4 (6.3)^f^	−0.47	18.2 (7.3)^g^	−0.34
	T3	21.9 (8.2)	16.1 (6.6)^i^	−0.78	17.3 (7.1)^j^	−0.60

^a^T1: baseline.

^b^N=39.

^c^Not applicable.

^d^N=35.

^e^T2: 4 weeks.

^f^N=34.

^g^N=32.

^h^T3: 8 weeks.

^i^N=37.

^j^N=33.

**Table 5:** Mean (SD) and effect size for quality of life, life satisfaction, and mindfulness scales.

Time of measurement		Control (n=38)	Full Intervention		Partial Intervention	
		Mean (SD)	Mean (SD)	Effect size	Mean (SD)	Effect size
**Quality of Life Scale** **16-item**						
	T1^a^	73.5 (16.4)	74.4 (12.4)^b^	N/A^c^	74.7 (12.9)^d^	N/A
	T2^e^	71.5 (15.8)	74.4 (14.2)^f^	0.19	75.4 (14.4)^g^	0.26
	T3^h^	69.7 (16.3)	78.2 (14)^i^	0.56	77.9 (14.1)^j^	0.54
**Brief Multidimensional Students’ Life Satisfaction Scale-** **Peabody Treatment Progress Battery** **6-item**						
	T1	3.5 (0.9)	3.6 (0.8)^b^	N/A	3.6 (0.8)^d^	N/A
	T2	3.5 (0.9)	3.7 (0.8)^f^	0.23	3.6 (0.9)^g^	0.11
	T3	3.3 (1)	3.8 (0.8)^i^	0.55	3.7 (0.9)^j^	0.42
**Five-Facet Mindfulness Questionnaire-Short Form** **24-item**						
	T1	76.8 (14.2)	71.4 (14.7)^b^	N/A	73.9 (9.9)^d^	N/A
	T2	76.6 (12.6)	76.6 (13)^f^	0.00	74.9 (10.8)^g^	−0.14
	T3	75.9 (15.6)	78.1 (15.4)^i^	0.14	78.5 (10.7)^j^	0.19
**Nonreact construct**						
	T1	15.4 (3.3)	13.3 (3.2)^b^	N/A	13.8 (4.3)^d^	N/A
	T2	14.9 (3.6)	14.5 (3)^f^	−0.12	14.5 (3.9)^g^	−0.11
	T3	14.6 (4.1)	15.5 (3.3)^i^	0.24	15.5 (4.3)^j^	0.21
**Observe construct**						
	T1	14.6 (3.7)	12.3 (3.8)^b^	N/A	13.2 (3.8)^d^	N/A
	T2	14.2 (3.4)	13.6 (2.9)^f^	−0.19	12.7 (3.4)^g^	−0.44
	T3	14.8 (3.8)	13.3 (3.4)^i^	−0.42	13.4 (3.1)^j^	−0.40
**Act aware construct**						
	T1	15.4 (4.6)	16.2 (5.2)^b^	N/A	15.5 (4.5)^d^	N/A
	T2	15.6 (4.9)	16.5 (3.9)^f^	0.20	16 (4.2)^g^	0.09
	T3	14.4 (5.5)	16.3 (4)^i^	0.39	16.6 (4.6)^j^	0.43
**Describe construct**						
	T1	16.5 (4.6)	15.4 (4.7)^b^	N/A	16.7 (3.7)^d^	N/A
	T2	16.4 (4.1)	16.6 (3.8)^f^	0.05	17.2 (3.7)^g^	0.20
	T3	16.7 (4.7)	17 (4.4)^i^	0.07	18 (4.1)^j^	0.29
**Judge construct**						
	T1	14.8 (4.3)	14.3 (4.5)^b^	N/A	14.7 (3.9)^d^	N/A
	T2	15.4 (4)	15.4 (4.6)^f^	0.00	14.4 (4.1)^g^	−0.25
	T3	15.6 (5.2)	16 (4.7)^i^	0.08	15.1 (4.3)^j^	−0.10

^a^T1: baseline.

^b^N=39.

^c^Not applicable.

^d^N=35.

^e^T2: 4 weeks.

^f^N=34.

^g^N=32.

^h^T3: 8 weeks.

^i^N=37.

^j^N=33.

### Results for Primary Outcomes

Table 6 shows the difference in the mean scores of the primary outcomes between T1 and T2 and T1 and T3 in the P-MVC and F-MVC groups compared with the WLC group. GEE method with AR(1) covariance structure was used, adjusting for potential confounding variables of age, gender, country of birth, paid work, unpaid work, self-rated health, weekly vigorous physical activities, and access to mental health private counseling via insurance.

In relation to *depression* in the F-MVC group compared with the WLC group, score reductions for PHQ-9 at T2 and T3 were statistically significant in both unadjusted (T2 unadjusted score change −2.47; *P=*.01; T3 unadjusted score change −3.39; *P<*.001) and adjusted (T2 adjusted score change −3.00; *P=*.015; T3 adjusted score-change −4.03; *P<*.001) analysis. The P-MVC group compared with the control group at T2 showed significant PHQ-9 score reduction on adjusted analysis (T2 adjusted score change −3.49; *P*=.01), whereas the difference was statistically significant at T3 in both unadjusted (T3 unadjusted score change −2.70; *P=*.01) and adjusted (T3 adjusted score change −4.82; *P*<.001) analysis. In relation to *anxiety* in the F-MVC group compared with the WLC group, there was no statistically significant score reduction for BAI at T2 or T3 in adjusted analysis (see details in Table 6). However, the effect of the P-MVC intervention on BAI score reduction reached statistical significance at T3 in adjusted analysis (T3 adjusted score change −7.35; *P=*.008). Compared with the WLC group, the F-MVC intervention also had a significant effect in reducing PSS stress score at both T2 in unadjusted analysis (T2 unadjusted score change −3.08; *P*=.015) and at T3 in unadjusted and adjusted analysis (T3 unadjusted score change −5.28; *P*<.001; T3 adjusted score change −5.32; *P*<.001). For the P-MVC, the PSS score reduction reached statistical significance only at T3 in unadjusted and adjusted analysis (T3 unadjusted score change −3.90; *P*=.009; T3 adjusted score change −5.61; *P*=.005).

**Table 6:** Generalized estimation equation with complete cases for score difference in depression, anxiety, and stress scales.

Score change at		Full intervention compared with control, mean score difference				Partial intervention compared with control, mean score difference			
		Unadjusted (SE)^a^	*P* value^b^	Adjusted^c^ (SE)	*P* value	Unadjusted (SE)	*P* value	Adjusted (SE)	*P* value
**Patient Health Questionnaire** **9-item**									
	T2^d^	−2.47 (0.98)	*.01*	−3.00 (1.21)	*.015*	−1.50 (1.06)	.16	−3.49 (1.38)	*.01*
	T3^e^	−3.39 (0.97)	*<.001*	−4.03 (1.20)	*<.001*	−2.70 (1.06)	*.01*	−4.82 (1.38)	*<.001*
**Beck Anxiety Inventory** **21-item**									
	T2	−2.18 (2.04)	.29	−0.54 (2.63)	.84	−2.62 (2.06)	.21	−5.45 (2.71)	.047
	T3	−4.82 (2.02)	*.019*	−3.21 (2.61)	.22	−4.22 (2.05)	.04	−7.35 (2.71)	*.008*
**Perceived Stress Scale 10-item**									
	T2	−3.08 (1.24)	*.015*	−3.06 (1.62)	.06	−1.91 (1.47)	.19	−3.52 (1.97)	.08
	T3	−5.28 (1.23)	*<.001*	−5.32 (1.60)	*<.001*	−3.90 (1.46)	*.009*	−5.61 (1.97)	*.005*

^a^SE of the mean score difference.

^b^*P* values <.02 are considered significant (shown in italic) to account for multiple comparisons.

^c^Adjusted for sex, age, country of birth, paid work, unpaid work, self-rated health, vigorous physical activities, and access to mental health private counseling via insurance.

^d^T2: 4 weeks.

^e^T3: 8 weeks.

### Results for Secondary Outcomes

Table 7 shows the score differences in the secondary outcomes between T1 and T2 and T1 and T3 in the P-MVC and F-MVC groups compared with the WLC group. Compared with the control group, changes in the QOLS score for quality of life at T3 showed statistically significant increase in unadjusted and adjusted analysis for both F-MVC (T3 unadjusted score change 8.67; *P*<.001; T3 adjusted score change 9.86; *P*<.001) and P-MVC groups (T3 unadjusted score change 7.21, *P*=.01; T3 adjusted score change 12.85, *P*<.001). The student life satisfaction measured by BMSLSS-PTPB showed statistically significant increase in the score only for the F-MVC group compared with the WLC group at T3 in unadjusted analysis (T3 unadjusted score change 2.69; *P*<.001). In terms of the level of mindfulness, FFMQ-SF scores improved when compared with controls in a statistically significant manner at T3 for F-MVC in unadjusted analysis (T3 score-change 7.8; *P*=.002) and for P-MVC in adjusted analysis (score change 6.83; *P*=.01).

**Table 7:** Generalized estimation equation with complete cases for score difference in quality of life, life satisfaction, and mindfulness scales

Score change at		Full intervention compared with control, mean score difference				Partial intervention compared with control, mean score difference			
		Unadjusted (SE)^a^	*P* value^b^	Adjusted^c^ (SE)	*P* value	Unadjusted (SE)	*P* value	Adjusted (SE)	*P* value
**Quality of Life Scale** **16-item**									
	T2^d^	3.04 (2.40)	.21	4.16 (2.86)	.15	3.16 (2.80)	.26	8.57 (3.81)	.03
	T3^e^	8.67 (2.37)	*<.001*	9.86 (2.83)	*<.001*	7.21 (2.76)	*.01*	12.85 (3.78)	*<.001*
**Brief Multidimensional Students’ Life Satisfaction Scale-** **Peabody Treatment Progress Battery** **6-item**									
	T2	1.34 (0.73)	.07	0.49 (1.03)	.64	0.28 (0.91)	.76	−0.08 (1.36)	.95
	T3	2.69 (0.73)	*<.001*	1.85 (1.02)	.07	1.66 (0.90)	.07	1.32 (1.35)	.33
**Five-Facet Mindfulness Questionnaire-Short Form** **24-item**									
	T2	4.96 (2.50)	.05	3.02 (3.26)	.36	−0.62 (1.98)	.75	1.50 (2.74)	.59
	T3	7.80 (2.48)	*.002*	6.02 (3.25)	.07	4.44 (1.95)	.03	6.83 (2.72)	*.01*

^a^SE of the mean score difference.

^b^*P* values <.02 are considered significant (shown in italic) to account for multiple comparisons.

^c^Adjusted for sex, age, country of birth, paid work, unpaid work, self-rated health, vigorous physical activities, and access to mental health private counseling via insurance.

^d^T2: 4 weeks.

^e^T3: 8 weeks.

### Academic Performance/Absenteeism and Use of Intervention

There was a statistically significant difference in the three groups at 8-week assessment in their self-perceived academic performance, *X^2^_4_*=13.6 (n=109); *P*=.008, and in the class absenteeism, *X^2^_4_*=17.2 (n=109); *P*=.002. For the academic performance, 32% (14/37) and 38% (11/34) of the students in the F-MVC and P-MVC groups, respectively, chose “seems better” in comparing current performance with what their performance was at the start of the study; the proportion was 8% (3/38) for “seems better” in the WLC group. For the class absenteeism, 19% (7/37) and 29% (10/34) of the students in the F-MVC and P-MVC groups, respectively, chose “less frequent,” whereas the proportion was 5% (2/38) in the WLC group.

In terms of the number of videos watched from “start to finish,” 65% (24/37) in the F-MVC group reported 7 to 12 videos for both the educational and mindfulness content. In the P-MVC group, 38% (13/34) reported watching 7 to 12 videos from “start to finish” for the educational content and 50% (17/34) for the mindfulness content. In response to this question, a handful chose “not applicable” in the F-MVC group (education 3/37, 8%; mindfulness 1/37, 3%) and in the P-MVC group (education 3/34, 9%; mindfulness 3/34, 9%), indicating nonuse by only a few participants. On comparing the two groups for watching less than 7 or greater than or equal to 7 videos from “start to finish,” the greater use of educational videos in the F-MVC group reached statistical significance, *X^2^_1_*=5.4 (n=65); *P*=.019. When asked about the “average frequency” of watching each video, the majority reported “one time” in both the F-MVC (education 24/37, 65%; mindfulness 17/37, 46%) and P-MVC (education 26/34, 77%; mindfulness 23/34, 68%) groups. Some used each video greater than or equal to 2 times in both the F-MVC (education 11/37, 30%; mindfulness 18/37, 49%) and P-MVC (education 5/34, 15%; mindfulness 8/34, 24%) groups. On group comparison for “average frequency” of using each video less than 2 or greater than or equal to 2 times, the more frequent use of mindfulness-practice videos in the F-MVC group reached statistical significance, *X^2^_1_*=4.5 (n=68); *P*=.033.

Participants in the F-MVC group also evaluated the discussion forums and videoconferencing. For the exchanges on discussion forums, participants “agreed” as to the appropriateness (mean 3.7, SD 0.77), supportiveness (mean 3.4, SD 0.82), and informativeness (mean 3.3, SD 0.77). There were 7 participants (7/27, 18.9%) who chose “not applicable” for questions on the discussion forum, indicating their nonuse. For the videoconferencing, participants “agreed” regarding its help in better understanding mindfulness practice and mental well-being (mean 3.8, SD 0.85), about help via direct message opportunity (mean 3.7, SD 1.0), as well as about ease in accessing the session (mean 3.7, SD 1.0), and session convenience (mean 3.5, SD 1.0). A total of 4 participants (4/37, 10.8%) chose “not applicable” for questions on the videoconferencing, indicating the absence of use.

## Discussion

### Principal Findings

The study investigated the efficacy of MVC, an 8-week internet-based mindfulness-CBT intervention aimed at reducing symptoms of anxiety, depression, and stress in undergraduate students. The Web-based full intervention, F-MVC, comprised 12 video-based modules with psychoeducational content and topically applied mindfulness practices that were released on alternate days over a 4-week period. This was followed by module access for additional 4 weeks. There were also peer-to-peer anonymous and asynchronous discussion forums for 4 weeks, and 20-min live videoconferences on mindfulness practice with a mental health professional on alternate days over the first 4 weeks. The partial intervention, P-MVC, comprised only the video-based modules, with the access schedule similar to the F-MVC intervention. Both forms of interventions were supported through email reminders over the initial 4 weeks, sent before the release of each module.

On testing, the F-MVC and the P-MVC interventions both significantly reduced scores for depression symptoms (PHQ-9), compared with the control group, at T3. The mean depression scores of participants generally reduced from the high end of subclinical depressive symptoms to the lower end. Within groups, the proportion of participants with scores of PHQ-9 ≥10 (a cutoff used to represent moderate-to-severe depression) at T3 reduced by 9% in the F-MVC and 16% in the P-MVC intervention groups. It is possible that the 8-week gains might be followed by additional positive change over the long term through continued practice of mindfulness and skill building; future research with longer follow-up periods would serve to examine such potential changes. The effects of both interventions on reducing the mean scores of perceived stress (PSS) were similar and statistically significant at T3, when compared with the controls. Another key finding of this study is that, on adjusted analysis, only the P-MVC intervention was effective in significantly reducing anxiety scores (BAI), compared with the controls, at T3, whereas the F-MVC intervention solely had a significant impact on BAI in the unadjusted analysis at T3. Although this finding could be due to inadequate sample power, the finding that the video intervention when combined with professional interactions (ie, discussion forum and videoconference) did *not* reduce anxieties, whereas the partial intervention (without professional contacts) did have a significant anxiety reduction effect may be instructive. Anxious subjects, avoidant of health professional contacts, might have responded more positively when assured that the entire program was Web-based and did not involve any “live” interactions. As the video and audio contacts apparently solely effectively reduced participant anxiety and depression levels, this finding has cost implications given that personnel costs often constitute the largest proportion of Web-based intervention costs. If the developed videos (with associated audios) are effective without the assistance of paid personnel, per participant costs may be reduced.

In terms of the secondary outcomes, both F-MVC and P-MVC interventions significantly increased quality of life scores at T3, compared with the controls, per adjusted analysis. The scores for the self-reported levels of mindfulness increased within both intervention groups at T3, but the group differences (in comparison with controls) were statistically significant only for the P-MVC group per adjusted analysis; the F-MVC group-associated results were statistically significant only in the unadjusted analysis. Among the five constructs of the FFMQ-SF scale, the *act aware* and *observe* subscales approached a moderate effect size of 0.4 at T3. The *describing* subscale had an effect size of 0.3 only in the P-MVC at T3. Another important finding was better self-reported academic performance and less absenteeism reported by the F-MVC and P-MVC respondents at T3, which was significantly different than the self-reports of the controls. Overall, the positive results for several of the examined secondary outcomes support the use of the MVC intervention in reducing depression, anxiety, and stress symptoms in an undergraduate student population.

### Strengths and Limitations

To the best of our knowledge, this is the first RCT of a Web-based interactive mindfulness-CBT program for college students in Canada. The study was implemented in a university setting with validated self-report standard instruments to measure the outcomes, completed by participants through online surveys to ensure accuracy and consistency in data collection. However, our recruitment was limited to a single institution, and the sample size was modest; both of these study elements warrant caution in interpreting and generalizing the results. Furthermore, the probability of reporting bias cannot be discounted due to the use of self-administered assessments, although such bias would be theoretically similar across study arms. We were unable to keep the participants blind to the intervention and control conditions once they opened the allocation envelopes after consenting. Another limitation is that two-third of the sample comprised female students, although female majority participation is a frequent finding in online study samples [38,41]. Nonetheless, our randomization worked, as gender was similarly distributed between the control and intervention groups (Table 2). In future research, stratification for gender would ensure more equal male-female samples. Future research with larger samples recruited from multiple universities and colleges would better test the generalizability of results. Another area for advancement is the collection of background use analytics, which was not built-in to our tested platform; although we gathered self-reported intervention use data that were encouraging.

### Comparison With Prior Work

Although mindfulness and CBT-based interventions have been reported as effective in reducing self-reported symptoms of anxiety and depression, few studies have investigated internet-based versions of such interventions. The results of our study are aligned with a handful of studies on Web-based mindfulness with student populations. For example, Nguyen et al [69] reported that their Web-based mindfulness intervention for students led to significant reductions in scores for depression, anxiety, and stress over time, although this was not significantly different from a group who received a Web-based general stress management intervention. Another Web-based mindfulness training program, which involved 8 weekly sessions with telephone support, also resulted in improved mental well-being, life satisfaction, energy, and reduced pain among students, although a pure control group was lacking [46]. Similarly, a brief Web-based mindfulness intervention by Cavanagh et al [48] was associated with significant reductions of scores for depression, anxiety, and perceived stress compared with a waitlist group, but the observed attrition rate was relatively high. Some studies have effectively used Web-based acceptance and commitment therapy [43,49] and found it effective in improving depression symptoms and psychological and physiological symptoms as well as associated with high levels of satisfaction. Likewise, in our study the life satisfaction scores statistically improved though only for the F-MVC group at T3. The findings of other studies and this study with students lend support to the effectiveness of Web-based mindfulness-CBT interventions for addressing common mental health disorders and promoting mental well-being among students.

The positive impacts of the studied intervention arms, F-MVC and P-MVC, on improving the academic performance and reducing the class absenteeism are noteworthy not only for the students themselves but for the academic institutions as well. These findings are consistent with emerging research on education and mindfulness that show increases in students’ focus on the task at hand and improved study habits and organization through a calmer view of their present situation [70,71]. Others have shown that mindfulness increases memory and concentration and reduces exam anxiety [72]. Given the difficulties experienced by youth entering postsecondary institutions, including students who drop out with long-term consequences, there is a need to advance further research and application of mindfulness-CBT tools, such as Web-based MVC.

The results of our study generate evidential support for CBT-informed mindfulness-based intervention in comparison with the control group, unlike other existing studies with students. The insights obtained about gains in mindfulness assessed with the FFMQ can contribute to the possible refinement of the MVC and other similar interventions. In our study, there was a noteworthy reduction in the FFMQ scores (when compared with controls) in the observation subscale in both the F-MVC and P-MVC arms at T2 and T3 (see Table 5). The observation subscale is largely associated with awareness of sensory-emotional experiences. Interestingly, the acting with awareness subscale, representing more generic instances of focal attention vs distractibility, increased in relation to controls. The increase in mean scores for the *acting with awareness* subscale is aligned with previous studies. For example, a longitudinal study with adolescents revealed that the *acting with awareness* subscale predicted a reduction in depression over time [73].

A unique feature of our study is testing both the full and partial MVC interventions. Findings supporting the reduction in anxiety symptoms among participant students who only used Web-based video modules offer a cost-effective way to address prevalent anxiety symptoms in postsecondary institutions. The student engagement process in our intervention is also noteworthy. Other Web-based mindfulness studies have revealed high attrition rates as a common problem, especially for those where the interactive methods used were limited [44-46,48,50]. Compared with these studies, we used more interactive methods to engage students, and this may have been effective in keeping attrition very low. Furthermore, only 8%, 19%, and 11% in the intervention groups chose “not applicable” when asked about their use of video-based modules, discussion forums, and videoconferencing, respectively. This suggests that a low number of participants did not access these intervention components. For wider use of Web-based programs among students, engagement strategies seem to be vital in ensuring optimal participation, retention, and completion for positive outcomes. With the widespread accessibility of internet and the evidence from literature including this study, Web-based mindfulness-CBT interventions such as MVC could effectively reduce symptoms of depression, anxiety, and stress among students and in a cost-efficient manner. Personal visits to a professional for mental health concerns are not the only economic burden on both users and the system; difficulties to access also exist for students because of mental health stigma and the challenges of commuting to and scheduling service delivery visits [16,17]. Our work informs the designing of appropriate programs accessed by students at their convenience, with some limited moderation by a mental health professional.

### Conclusions

The study demonstrated the effectiveness of an internet-based mindfulness-CBT intervention in reducing depression, anxiety, and stress symptoms among students. The student-centered design of the platform, which included design features identified through focus groups, might have contributed to the positive impact and reduced attrition. Further studies with larger samples are needed to enhance the generalizability of study results. In addition, larger samples are likely to enhance understanding from the perspective of clinical recovery by examining the number of individuals who experience a shift from the moderate or severe levels of depression or anxiety to lower levels and enhanced functioning. Nonetheless, current findings suggest that Web-based mindfulness-CBT interventions, such as the one studied here, offer a good opportunity to address common mental health conditions in a postsecondary population while simultaneously reducing the burden on traditional counseling and services.

## Appendix

Multimedia Appendix 1 Generalized estimation equation with last observation carried forward for score difference in depression, anxiety, and stress scales.

Multimedia Appendix 2 Generalized estimation equation with last observation carried forward for score difference in quality of life, life satisfaction, and mindfulness scales.

Multimedia Appendix 3 CONSORT-EHEALTH checklist (V 1.6.1).

## Abbreviations

BAI: Beck Anxiety Inventory
BMSLSS-PTPB: Brief Multidimensional Students’ Life Satisfaction Scale-Peabody Treatment Progress Battery
CBT: cognitive behavioral therapy
CIHR: Canadian Institutes for Health Research
FFMQ-SF: Five-Facet Mindfulness Questionnaire-Short Form
F-MVC: full Mindfulness Virtual Community
GEE: generalized estimation equation
LOCF: last observation carried forward
MBCT: mindfulness-based cognitive therapy
MVC: Mindfulness Virtual Community
PHQ-9: Patient Health Questionnaire
P-MVC: partial Mindfulness Virtual Community
PSS: Perceived Stress Scale
QOLS: Quality of Life Scale
RCT: randomized controlled trial
WLC: waitlist control

## References

[1] Turner JC, Keller A (2015). College health surveillance network: epidemiology and health care utilization of college students at US 4-year universities. J Am Coll Health.

[2] Oswalt SB, Lederer AM, Chestnut-Steich K, Day C, Halbritter A, Ortiz D (2020). Trends in college students' mental health diagnoses and utilization of services, 2009-2015. J Am Coll Health.

[3] Lipson SK, Lattie EG, Eisenberg D (2019). Increased rates of mental health service utilization by US College students: 10-year population-level trends (2007-2017). Psychiatr Serv.

[4] Lipson SK, Gaddis SM, Heinze J, Beck K, Eisenberg D (2015). Variations in student mental health and treatment utilization across US colleges and universities. J Am Coll Health.

[5] Lipson SK, Zhou S, Wagner B, Beck K, Eisenberg D (2016). Major differences: variations in undergraduate and graduate student mental health and treatment utilization across academic disciplines. J College Stud Psychother.

[6] Healthy Minds Network.

[7] Substance Use and Mental Health Services Administration.

[8] Twenge JM, Cooper AB, Joiner TE, Duffy ME, Binau SG (2019). Age, period, and cohort trends in mood disorder indicators and suicide-related outcomes in a nationally representative dataset, 2005-2017. J Abnorm Psychol.

[9] Chernomas WM, Shapiro C (2013). Stress, depression, and anxiety among undergraduate nursing students. Int J Nurs Educ Scholarsh.

[10] Schlarb AA, Claßen M, Grünwald J, Vögele C (2017). Sleep disturbances and mental strain in university students: results from an online survey in Luxembourg and Germany. Int J Ment Health Syst.

[11] Moutinho IL, Maddalena ND, Roland RK, Lucchetti AL, Tibiriçá SH, Ezequiel OD, Lucchetti G (2017). Depression, stress and anxiety in medical students: a cross-sectional comparison between students from different semesters. Rev Assoc Med Bras (1992).

[12] Cheung T, Wong SY, Wong KY, Law LY, Ng K, Tong MT, Wong KY, Ng MY, Yip PS (2016). Depression, anxiety and symptoms of stress among baccalaureate nursing students in Hong Kong: a cross-sectional study. Int J Environ Res Public Health.

[13] Bayram N, Bilgel N (2008). The prevalence and socio-demographic correlations of depression, anxiety and stress among a group of university students. Soc Psychiatry Psychiatr Epidemiol.

[14] Kulsoom B, Afsar NA (2015). Stress, anxiety, and depression among medical students in a multiethnic setting. Neuropsychiatr Dis Treat.

[15] Downs N, Galles E, Skehan B, Lipson SK (2018). Be true to our schools-models of care in college mental health. Curr Psychiatry Rep.

[16] Sareen J, Jagdeo A, Cox BJ, Clara I, ten Have M, Belik S, de Graaf Ron, Stein MB (2007). Perceived barriers to mental health service utilization in the United States, Ontario, and the Netherlands. Psychiatr Serv.

[17] Pedersen ER, Paves AP (2014). Comparing perceived public stigma and personal stigma of mental health treatment seeking in a young adult sample. Psychiatry Res.

[18] Lees J, Dietsche P (2012). Centre for Innovation in Campus Mental Health.

[19] Galagher RP (2014). D-Scholarship@Pitt - University of Pittsburgh.

[20] Kabat‐Zinn J (2003). Mindfulness‐based interventions in context: past, present, and future. Clin Psychol.

[21] Kabat-Zinn J (1982). An outpatient program in behavioral medicine for chronic pain patients based on the practice of mindfulness meditation: theoretical considerations and preliminary results. Gen Hosp Psychiatry.

[22] Keng S, Smoski MJ, Robins CJ (2011). Effects of mindfulness on psychological health: a review of empirical studies. Clin Psychol Rev.

[23] Segal ZV, Williams JM, Teasdale JD (2002). Mindfulness-Based Cognitive Therapy for Depression: A New Approach to Preventing Relapse.

[24] Brown KW, Ryan RM (2003). The benefits of being present: mindfulness and its role in psychological well-being. J Pers Soc Psychol.

[25] Grossman P, Niemann L, Schmidt S, Walach H (2004). Mindfulness-based stress reduction and health benefits. A meta-analysis. J Psychosom Res.

[26] Chiesa A, Serretti A (2009). Mindfulness-based stress reduction for stress management in healthy people: a review and meta-analysis. J Altern Complement Med.

[27] Hofmann SG, Sawyer AT, Witt AA, Oh D (2010). The effect of mindfulness-based therapy on anxiety and depression: a meta-analytic review. J Consult Clin Psychol.

[28] Vøllestad J, Nielsen MB, Nielsen GH (2012). Mindfulness- and acceptance-based interventions for anxiety disorders: a systematic review and meta-analysis. Br J Clin Psychol.

[29] Eberth J, Sedlmeier P (2012). The effects of mindfulness meditation: a meta-analysis. Mindfulness.

[30] Sedlmeier P, Eberth J, Schwarz M, Zimmermann D, Haarig F, Jaeger S, Kunze S (2012). The psychological effects of meditation: a meta-analysis. Psychol Bull.

[31] Yang E, Schamber E, Meyer RM, Gold JI (2018). Happier Healers: randomized controlled trial of mobile mindfulness for stress management. J Altern Complement Med.

[32] Falsafi N (2016). A randomized controlled trial of mindfulness versus yoga: effects on depression and/or anxiety in college students. J Am Psychiatr Nurses Assoc.

[33] Dvořáková K, Kishida M, Li J, Elavsky S, Broderick PC, Agrusti MR, Greenberg MT (2017). Promoting healthy transition to college through mindfulness training with first-year college students: Pilot randomized controlled trial. J Am Coll Health.

[34] McIndoo CC, File AA, Preddy T, Clark CG, Hopko DR (2016). Mindfulness-based therapy and behavioral activation: a randomized controlled trial with depressed college students. Behav Res Ther.

[35] Gallego J, Aguilar-Parra JM, Cangas AJ, Langer ÁI, Mañas I (2015). Effect of a mindfulness program on stress, anxiety and depression in university students. Span J Psychol.

[36] Song Y, Lindquist R (2015). Effects of mindfulness-based stress reduction on depression, anxiety, stress and mindfulness in Korean nursing students. Nurse Educ Today.

[37] Warnecke E, Quinn S, Ogden K, Towle N, Nelson MR (2011). A randomised controlled trial of the effects of mindfulness practice on medical student stress levels. Med Educ.

[38] Chen Y, Yang X, Wang L, Zhang X (2013). A randomized controlled trial of the effects of brief mindfulness meditation on anxiety symptoms and systolic blood pressure in Chinese nursing students. Nurse Educ Today.

[39] van der Riet P, Levett-Jones T, Aquino-Russell C (2018). The effectiveness of mindfulness meditation for nurses and nursing students: an integrated literature review. Nurse Educ Today.

[40] McConville J, McAleer R, Hahne A (2017). Mindfulness training for health profession students-the effect of mindfulness training on psychological well-being, learning and clinical performance of health professional students: a systematic review of randomized and non-randomized controlled trials. Explore (NY).

[41] O'Driscoll M, Byrne S, Mc Gillicuddy A, Lambert S, Sahm LJ (2017). The effects of mindfulness-based interventions for health and social care undergraduate students - a systematic review of the literature. Psychol Health Med.

[42] Guillaumie L, Boiral O, Champagne J (2017). A mixed-methods systematic review of the effects of mindfulness on nurses. J Adv Nurs.

[43] Räsänen P, Lappalainen P, Muotka J, Tolvanen A, Lappalainen R (2016). An online guided ACT intervention for enhancing the psychological wellbeing of university students: A randomized controlled clinical trial. Behav Res Ther.

[44] Antonson C, Thorsén F, Sundquist J, Sundquist K (2018). Upper secondary school students' compliance with two internet-based self-help programmes: a randomised controlled trial. Eur Child Adolesc Psychiatry.

[45] Noone C, Hogan MJ (2018). A randomised active-controlled trial to examine the effects of an online mindfulness intervention on executive control, critical thinking and key thinking dispositions in a university student sample. BMC Psychol.

[46] Mak WW, Chio FH, Chan AT, Lui WW, Wu EK (2017). The efficacy of internet-based mindfulness training and cognitive-behavioral training with telephone support in the enhancement of mental health among college students and young working adults: randomized controlled trial. J Med Internet Res.

[47] Aspy DJ, Proeve M (2017). Mindfulness and loving-kindness meditation. Psychol Rep.

[48] Cavanagh K, Strauss C, Cicconi F, Griffiths N, Wyper A, Jones F (2013). A randomised controlled trial of a brief online mindfulness-based intervention. Behav Res Ther.

[49] Lappalainen P, Langrial S, Oinas-Kukkonen H, Tolvanen A, Lappalainen R (2015). Web-based acceptance and commitment therapy for depressive symptoms with minimal support: a randomized controlled trial. Behav Modif.

[50] Mak WW, Chan AT, Cheung EY, Lin CL, Ngai KC (2015). Enhancing web-based mindfulness training for mental health promotion with the health action process approach: randomized controlled trial. J Med Internet Res.

[51] Olthuis JV, Watt MC, Bailey K, Hayden JA, Stewart SH (2015). Therapist-supported internet cognitive behavioural therapy for anxiety disorders in adults. Cochrane Database Syst Rev.

[52] Webb CA, Rosso IM, Rauch SL (2017). Internet-based cognitive-behavioral therapy for depression: current progress and future directions. Harv Rev Psychiatry.

[53] Rickwood DJ, Mazzer KR, Telford NR (2015). Social influences on seeking help from mental health services, in-person and online, during adolescence and young adulthood. BMC Psychiatry.

[54] El Morr C, Maule C, Ashfaq I, Ritvo P, Ahmad F (2017). A student-centered mental health virtual community needs and features: a focus group study. Stud Health Technol Inform.

[55] Ahmad F, El Morr C, Ritvo P (2018). Mindfulness Virtual Community for Student Mental Health. Proceedings of the Center for innovation in Campus Health Conference 2018.

[56] Ahmad F, Wang JJ, El Morr C, El Morr C (2017). Online mindfulness interventions: a systematic review. Novel Applications of Virtual Communities in Healthcare Settings.

[57] Azam MA, Mongrain M, Vora K, Pirbaglou M, Azargive S, Changoor T, Wayne N, Guglietti C, Macpherson A, Irvine J, Rotondi M, Smith D, Perez D, Ritvo P (2016). Mindfulness as an alternative for supporting university student mental health: Cognitive-emotional and depressive self-criticism measures. Int J Educ Psychol.

[58] Radhu N, Daskalakis ZJ, Arpin-Cribbie CA, Irvine J, Ritvo P (2012). Evaluating a web-based cognitive-behavioral therapy for maladaptive perfectionism in university students. J Am Coll Health.

[59] Boutron I, Altman DG, Moher D, Schulz KF, Ravaud P, CONSORT NPT Group (2017). CONSORT Statement for randomized trials of nonpharmacologic treatments: a 2017 update and a CONSORT extension for nonpharmacologic trial abstracts. Ann Intern Med.

[60] Eysenbach G, CONSORT-EHEALTH Group (2011). CONSORT-EHEALTH: improving and standardizing evaluation reports of Web-based and mobile health interventions. J Med Internet Res.

[61] Doig GS, Simpson F (2005). Randomization and allocation concealment: a practical guide for researchers. J Crit Care.

[62] Spitzer RL, Kroenke K, Williams JB (1999). Validation and utility of a self-report version of PRIME-MD: the PHQ primary care study. Primary Care Evaluation of Mental Disorders. Patient Health Questionnaire. J Am Med Assoc.

[63] Beck AT, Epstein N, Brown G, Steer RA (1988). An inventory for measuring clinical anxiety: psychometric properties. J Consult Clin Psychol.

[64] Cohen S, Kamarck T, Mermelstein R (1983). A global measure of perceived stress. J Health Soc Behav.

[65] Flanagan JC (1982). Measurement of quality of life: current state of the art. Arch Phys Med Rehabil.

[66] Seligson J, Huebner ES, Valois RF (2003). Preliminary validation of the Brief Multidimensional Students' Life Satisfaction Scale (BMSLSS). Soc Indic Res.

[67] Bohlmeijer E, ten Klooster PM, Fledderus M, Veehof M, Baer R (2011). Psychometric properties of the five facet mindfulness questionnaire in depressed adults and development of a short form. Assessment.

[68] Hedeker D, Gibbons RD, Waternaux C (1999). Sample size estimation for longitudinal designs with attrition: comparing time-related contrasts between two groups. J Edu Behav Stat.

[69] Nguyen-Feng VN, Greer CS, Frazier P (2017). Using online interventions to deliver college student mental health resources: evidence from randomized clinical trials. Psychol Serv.

[70] Rodgers L (2014). A calmer happier kid. Scholastic Parent Child.

[71] Broderick PC, Jennings PA (2012). Mindfulness for adolescents: a promising approach to supporting emotion regulation and preventing risky behavior. New Dir Youth Dev.

[72] Docksai R (2013). A mindful approach to learning: new research shows potential for 'Mindfulness training' to boost student productivity. Futurist.

[73] Royuela-Colomer E, Calvete E (2016). Mindfulness facets and depression in adolescents: rumination as a mediator. Mindfulness.

