# Supplementary material for: An Eight-Week, Web-Based Mindfulness Virtual Community Intervention for Students’ Mental Health: Randomized Controlled Trial
Source: JMIR Ment Health. 2020 Feb 18;7(2):e15520. doi: 10.2196/15520 (PMC7055779; doi:10.2196/15520)
Supplement: Multimedia Appendix 1 [file mental_v7i2e15520_app1.docx]

**Appendix 1: Generalized estimation equation with last observation carried forward for score difference in depression, anxiety, and stress scales.**

| Score change at | | Full intervention compared with control, mean score difference | | | | Partial intervention compared with control, mean score difference | | | |
| --- | --- | --- | --- | --- | --- | --- | --- | --- | --- |
|  | Unadjusted (SE)^a^ | | P value^b^ | Adjusted^c^ (SE) | P value | Unadjusted (SE) | P value | Adjusted (SE) | P value |
| **Patient Health Questionnaire 9-item** | | | | | | | | | |
| T2^d^ | -2.62 (0.94) | | *.01* | -3.24 (1.19) | *.01* | -1.40 (1.04) | .18 | -3.17 (1.35) | .02 |
| T3^e^ | -3.43 (0.94) | | *<.001* | -4.06 (1.19) | *<.001* | -2.50 (1.04) | *.017* | -4.28 (1.35) | *<.01* |
| **Beck Anxiety Inventory 21-item** | | | | | | | | | |
| T2 | -2.31 (1.96) | | .24 | -0.68 (2.50) | .79 | -2.64 (2.00) | .19 | -5.04 (2.63) | .06 |
| T3 | -4.85 (1.96) | | *.015* | -3.22 (2.50) | .20 | -3.91 (2.00) | .05 | -6.31 (2.63) | *.018* |
| **Perceived Stress Scale 10-item** | | | | | | | | | |
| T2 | -3.34 (1.20) | | *.01* | -3.34 (1.56) | .03 | -1.88 (1.41) | .19 | -3.29 (1.84) | .08 |
| T3 | -5.29 (1.20) | | *<.001* | -5.29 (1.56) | *<.001* | -3.68 (1.41) | *.01* | -5.08 (1.84) | *.01* |

*^a^ Standard error of the mean score difference.*

*^b^ P values <.02 are considered significant (shown with italic) to account for multiple comparisons.*

*^c^ Adjusted for sex, age, country of birth, paid work, unpaid work, self-rated health, vigorous physical activities, and access to mental health private counselling via insurance.*

*^d^ T2: 4 weeks.*

*^e^ T3: 8 weeks.*
