# Supplementary material for: An Eight-Week, Web-Based Mindfulness Virtual Community Intervention for Students’ Mental Health: Randomized Controlled Trial
Source: JMIR Ment Health. 2020 Feb 18;7(2):e15520. doi: 10.2196/15520 (PMC7055779; doi:10.2196/15520)
Supplement: Multimedia Appendix 2 [file mental_v7i2e15520_app2.docx]

**Appendix 2: Generalized estimation equation with last observation carried forward for score difference in quality of life, life satisfaction, and mindfulness scales.**

| Score change at | Full intervention compared with control, mean score difference | | | | Partial intervention compared with control, mean score difference | | | |
| --- | --- | --- | --- | --- | --- | --- | --- | --- |
|  | Unadjusted (SE)^a^ | P value^d^ | Adjusted^b^ (SE) | P value^c^ | Unadjusted (SE) | P value | Adjus^ted^ (SE) | P value |
| **Quality of Life Scale** **16-item** | | | | | | | | |
| T2^d^ | 3.18 (2.27) | .17 | 4.62 (2.73) | .09 | 3.45 (2.64) | .19 | 8.07 (3.58) | .03 |
| T3^e^ | 8.56 (2.27) | *<.001* | 10.00 (2.73) | *<.001* | 7.00 (2.64) | *.01* | 11.63 (3.58) | *<.001* |
| **Brief Multidimensional Students’ Life Satisfaction Scale-Peabody Treatment Progress Battery 6-item** | | | | | | | | |
| T2 | 1.25 (0.70) | .08 | 0.50 (.98) | .61 | 0.30 (0.86) | .73 | -0.14 (1.26) | .91 |
| T3 | 2.66 (0.70) | *<.001* | 1.91 (.98) | .05 | 1.62 (0.86) | .06 | 1.18 (1.26) | .35 |
| **Five-Facet Mindfulness Questionnaire-Short Form** **24-item** | | | | | | | | |
| T2 | 4.71 (2.32) | .05 | 2.87 (3.02) | .34 | -0.64 (1.89) | .74 | 0.89 (2.56) | .73 |
| T3 | 7.51 (2.32) | *.01* | 5.67 (3.02) | .06 | 4.18 (1.89) | .03 | 5.71 (2.56) | .03 |
| *^a^ Standard error of the mean score difference.*  *^b^ P values <.02 are considered significant (shown with italic) to account for multiple comparisons.*  *^c^ Adjusted for sex, age, country of birth, paid work, unpaid work, self-rated health, vigorous physical activities, and access to mental health private counselling via insurance.*  *^d^ T2: 4 weeks.*  *^e^ T3: 8 weeks.* | | | | | | | | |
