# Supplementary material for: An Eight-Week, Web-Based Mindfulness Virtual Community Intervention for Students’ Mental Health: Randomized Controlled Trial
Source: JMIR Ment Health. 2020 Feb 18;7(2):e15520. doi: 10.2196/15520 (PMC7055779; doi:10.2196/15520)
Supplement: Multimedia Appendix 3 [file mental_v7i2e15520_app3.pdf]

CONSORT-EHEALTH (V 1.6.1) - Submission/Publication Form

The CONSORT-EHEALTH checklist is intended for authors of randomized trials evaluating web-based and Internet-based applications/interventions, including mobile interventions, electronic games (incl multiplayer games), social media, certain telehealth applications, and other interactive and/or networked electronic applications. Some of the items (e.g. all subitems under item 5 - description of the intervention) may also be applicable for other study designs.

The goal of the CONSORT EHEALTH checklist and guideline is to be
a) a guide for reporting for authors of RCTs,
b) to form a basis for appraisal of an ehealth trial (in terms of validity)

CONSORT-EHEALTH items/subitems are MANDATORY reporting items for studies published in the Journal of Medical Internet Research and other journals / scientific societies endorsing the checklist.

Items numbered 1., 2., 3., 4a., 4b etc are original CONSORT or CONSORT-NPT (non-pharmacologic treatment) items.
Items with Roman numerals (i., ii, iii, iv etc.) are CONSORT-EHEALTH extensions/clarifications.

As the CONSORT-EHEALTH checklist is still considered in a formative stage, we would ask that you also RATE ON A SCALE OF 1-5 how important/useful you feel each item is FOR THE PURPOSE OF THE CHECKLIST and reporting guideline (optional).

Mandatory reporting items are marked with a red *.
In the textboxes, either copy & paste the relevant sections from your manuscript into this form - please include any quotes from your manuscript in QUOTATION MARKS, or answer directly by providing additional information not in the manuscript, or elaborating on why the item was not relevant for this study.

YOUR ANSWERS WILL BE PUBLISHED AS A SUPPLEMENTARY FILE TO YOUR PUBLICATION IN JMIR AND ARE CONSIDERED PART OF YOUR PUBLICATION (IF ACCEPTED).
Please fill in these questions diligently. Information will not be copyedited, so please use proper spelling and grammar, use correct capitalization, and avoid abbreviations.

DO NOT FORGET TO SAVE AS PDF _AND_ CLICK THE SUBMIT BUTTON SO YOUR ANSWERS ARE IN OUR DATABASE !!!

Citation Suggestion (if you append the pdf as Appendix we suggest to cite this paper in the caption):
Eysenbach G, CONSORT-EHEALTH Group
CONSORT-EHEALTH: Improving and Standardizing Evaluation Reports of Web-based and Mobile Health Interventions
J Med Internet Res 2011;13(4):e126
URL: http://www.jmir.org/2011/4/e126/
doi: 10.2196/jmir.1923
PMID: 22209829

* Required

Your name *

First Last

Farah Ahmad

Primary Affiliation (short), City, Country *

University of Toronto, Toronto, Canada

York University, Toronto, Canada

Your e-mail address *

abc@gmail.com

farahmad@yorku.ca

Title of your manuscript *

Provide the (draft) title of your manuscript.

An Eight-Week, Web-Based Mindfulness Virtual Community Intervention for Students' Mental Health: Randomized Controlled Trial

Name of your App/Software/Intervention *

If there is a short and a long/alternate name, write the short name first and add the long name in brackets.

MVC (Mindfulness Virtual Community)

Evaluated Version (if any)

e.g. "V1", "Release 2017-03-01", "Version 2.0.27913"

Not applicable

Language(s) *

What language is the intervention/app in? If multiple languages are available, separate by comma (e.g. "English, French")

English

URL of your Intervention Website or App

e.g. a direct link to the mobile app on app in appstore (itunes, Google Play), or URL of the website. If the intervention is a DVD or hardware, you can also link to an Amazon page.

http://www.studentsmentalhealth.com/

URL of an image/screenshot (optional)

Your answer

Accessibility *

Can an enduser access the intervention presently?

- ○ access is free and open
- ○ access only for special usergroups, not open
- ○ access is open to everyone, but requires payment/subscription/in-app purchases
- ○ app/intervention no longer accessible
- ◉ Other: access of modules could be discussed with the authors

Primary Medical Indication/Disease/Condition *

e.g. "Stress", "Diabetes", or define the target group in brackets after the condition, e.g. "Autism (Parents of children with)", "Alzheimers (Informal Caregivers of)"

Depression, anxiety & stress symptoms (un

Primary Outcomes measured in trial *

comma-separated list of primary outcomes reported in the trial

Depression, anxiety, stress

Secondary/other outcomes

Are there any other outcomes the intervention is expected to affect?

Quality of life, life satisfaction, mindfulness, academic performance, class attendance/ absenteeism, use of MVC

Recommended "Dose" *

What do the instructions for users say on how often the app should be used?

- ( ) Approximately Daily
- ( ) Approximately Weekly
- ( ) Approximately Monthly
- ( ) Approximately Yearly
- ( ) "as needed"
- (•) Other: ad libitum

Approx. Percentage of Users (starters) still using the app as recommended after 3 months *

- ○ unknown / not evaluated
- ○ 0-10%
- ○ 11-20%
- ○ 21-30%
- ○ 31-40%
- ○ 41-50%
- ○ 51-60%
- ○ 61-70%
- ○ 71%-80%
- ○ 81-90%
- ○ 91-100%
- ◉ Other: Participants did not have access after the 8 week study period

Overall, was the app/intervention effective? *

- (•) yes: all primary outcomes were significantly better in intervention group vs control
- ( ) partly: SOME primary outcomes were significantly better in intervention group vs control
- ( ) no statistically significant difference between control and intervention
- ( ) potentially harmful: control was significantly better than intervention in one or more outcomes
- ( ) inconclusive: more research is needed
- ( ) Other:

Article Preparation Status/Stage *

At which stage in your article preparation are you currently (at the time you fill in this form)

- ( ) not submitted yet - in early draft status
- ( ) not submitted yet - in late draft status, just before submission
- ( ) submitted to a journal but not reviewed yet
- ( ) submitted to a journal and after receiving initial reviewer comments
- (•) submitted to a journal and accepted, but not published yet
- ( ) published
- ( ) Other:

Journal *

If you already know where you will submit this paper (or if it is already submitted), please provide the journal name (if it is not JMIR, provide the journal name under "other")

- ( ) not submitted yet / unclear where I will submit this
- ( ) Journal of Medical Internet Research (JMIR)
- ( ) JMIR mHealth and UHealth
- ( ) JMIR Serious Games
- (x) JMIR Mental Health
- ( ) JMIR Public Health
- ( ) JMIR Formative Research
- ( ) Other JMIR sister journal
- ( ) Other:

Is this a full powered effectiveness trial or a pilot/feasibility trial? *

- (x) Pilot/feasibility
- ( ) Fully powered

Manuscript tracking number *

If this is a JMIR submission, please provide the manuscript tracking number under "other" (The ms tracking number can be found in the submission acknowledgement email, or when you login as author in JMIR. If the paper is already published in JMIR, then the ms tracking number is the four-digit number at the end of the DOI, to be found at the bottom of each published article in JMIR)

- ○ no ms number (yet) / not (yet) submitted to / published in JMIR
- ◉ Other: 15520

TITLE AND ABSTRACT

1a) TITLE: Identification as a randomized trial in the title

1a) Does your paper address CONSORT item 1a? *

I.e does the title contain the phrase "Randomized Controlled Trial"? (if not, explain the reason under "other")

- ◉ yes
- ○ Other:

1a-i) Identify the mode of delivery in the title

Identify the mode of delivery. Preferably use “web-based” and/or “mobile” and/or “electronic game” in the title. Avoid ambiguous terms like “online”, “virtual”, “interactive”. Use “Internet-based” only if Intervention includes non-web-based Internet components (e.g. email), use “computer-based” or “electronic” only if offline products are used. Use “virtual” only in the context of “virtual reality” (3-D worlds). Use “online” only in the context of “online support groups”. Complement or substitute product names with broader terms for the class of products (such as “mobile” or “smart phone” instead of “iphone”), especially if the application runs on different platforms.

	1	2	3	4	5	
subitem not at all important	○	○	○	○	◉	essential

Does your paper address subitem 1a-i? *

Copy and paste relevant sections from manuscript title (include quotes in quotation marks "like this" to indicate direct quotes from your manuscript), or elaborate on this item by providing additional information not in the ms, or briefly explain why the item is not applicable/relevant for your study

Like this "Web-based"

1a-ii) Non-web-based components or important co-interventions in title

Mention non-web-based components or important co-interventions in title, if any (e.g., “with telephone support”).

	1	2	3	4	5	
subitem not at all important	○	◉	○	○	○	essential

Does your paper address subitem 1a-ii?

Copy and paste relevant sections from manuscript title (include quotes in quotation marks "like this" to indicate direct quotes from your manuscript), or elaborate on this item by providing additional information not in the ms, or briefly explain why the item is not applicable/relevant for your study

All co-interventions were delivered via the web-based platform

1a-iii) Primary condition or target group in the title

Mention primary condition or target group in the title, if any (e.g., “for children with Type I Diabetes”) Example: A Web-based and Mobile Intervention with Telephone Support for Children with Type I Diabetes: Randomized Controlled Trial

	1	2	3	4	5	
subitem not at all important	○	○	○	○	◉	essential

Does your paper address subitem 1a-iii? *

Copy and paste relevant sections from manuscript title (include quotes in quotation marks "like this" to indicate direct quotes from your manuscript), or elaborate on this item by providing additional information not in the ms, or briefly explain why the item is not applicable/relevant for your study

"Students' Mental Health"

1b) ABSTRACT: Structured summary of trial design, methods, results, and conclusions

NPT extension: Description of experimental treatment, comparator, care providers, centers, and blinding status.

1b-i) Key features/functionalities/components of the intervention and comparator in the METHODS section of the ABSTRACT

Mention key features/functionalities/components of the intervention and comparator in the abstract. If possible, also mention theories and principles used for designing the site. Keep in mind the needs of systematic reviewers and indexers by including important synonyms. (Note: Only report in the abstract what the main paper is reporting. If this information is missing from the main body of text, consider adding it)

	1	2	3	4	5	
subitem not at all important	○	○	○	○	◉	essential

Does your paper address subitem 1b-i? *

Copy and paste relevant sections from the manuscript abstract (include quotes in quotation marks "like this" to indicate direct quotes from your manuscript), or elaborate on this item by providing additional information not in the ms, or briefly explain why the item is not applicable/relevant for your study

The intervention features/functionalities/components are described like this "The first 4 weeks of the full MVC intervention (F-MVC) comprised: (1) 12 video-based modules with psycho-education on students' preidentified stressful topics and topically applied mindfulness practice; (2) anonymous peer-to-peer discussion forums; and (3) anonymous, group-based, professionally guided, 20-min live videoconferences. The second 4 weeks of F-MVC involved access only to video-based modules. The 8-week partial MVC (P-MVC) comprised 12 video-based modules."

The comparator is described like this "A randomized controlled trial was conducted with 4 parallel arms: F-MVC, P-MVC, waitlist control (WLC), and group-based face-to-

1b-ii) Level of human involvement in the METHODS section of the ABSTRACT

Clarify the level of human involvement in the abstract, e.g., use phrases like “fully automated” vs. “therapist/nurse/care provider/physician-assisted” (mention number and expertise of providers involved, if any). (Note: Only report in the abstract what the main paper is reporting. If this information is missing from the main body of text, consider adding it)

	1	2	3	4	5	
subitem not at all important	○	○	◉	○	○	essential

Does your paper address subitem 1b-ii?

Copy and paste relevant sections from the manuscript abstract (include quotes in quotation marks "like this" to indicate direct quotes from your manuscript), or elaborate on this item by providing additional information not in the ms, or briefly explain why the item is not applicable/relevant for your study

The word count limit for Abstract is a challenge to add so much detail. We tried to add some of it like this "The first 4 weeks of the full MVC intervention (F-MVC) comprised: (1) 12 video-based modules with psycho-education on students’ preidentified stressful topics and topically applied mindfulness practice; (2) anonymous peer-to-peer discussion forums; and (3) anonymous, group-based, professionally guided, 20-min live videoconferences. The second 4 weeks of F-MVC

1b-iii) Open vs. closed, web-based (self-assessment) vs. face-to-face assessments in the METHODS section of the ABSTRACT

Mention how participants were recruited (online vs. offline), e.g., from an open access website or from a clinic or a closed online user group (closed usergroup trial), and clarify if this was a purely web-based trial, or there were face-to-face components (as part of the intervention or for assessment). Clearly say if outcomes were self-assessed through questionnaires (as common in web-based trials). Note: In traditional offline trials, an open trial (open-label trial) is a type of clinical trial in which both the researchers and participants know which treatment is being administered. To avoid confusion, use “blinded” or “unblinded” to indicated the level of blinding instead of “open”, as “open” in web-based trials usually refers to “open access” (i.e. participants can self-enrol). (Note: Only report in the abstract what the main paper is reporting. If this information is missing from the main body of text, consider adding it)

	1	2	3	4	5	
subitem not at all important	○	○	○	○	◉	essential

Does your paper address subitem 1b-iii?

Copy and paste relevant sections from the manuscript abstract (include quotes in quotation marks "like this" to indicate direct quotes from your manuscript), or elaborate on this item by providing additional information not in the ms, or briefly explain why the item is not applicable/relevant for your study

Like this "Students recruited through multiple strategies consented and were randomized: WLC=40; F-MVC=40, P-MVC=39; all learned about allocation after consenting." We could not use the exact wording of "blinded" in the Abstract as students and research assistant were blind only up to the stage of consent provision. The main paper to describe the full context and uses the word "blinded'.

1b-iv) RESULTS section in abstract must contain use data

Report number of participants enrolled/assessed in each group, the use/uptake of the intervention (e.g., attrition/adherence metrics, use over time, number of logins etc.), in addition to primary/secondary outcomes. (Note: Only report in the abstract what the main paper is reporting. If this information is missing from the main body of text, consider adding it)

	1	2	3	4	5	
subitem not at all important	○	○	○	○	◉	essential

Does your paper address subitem 1b-iv?

Copy and paste relevant sections from the manuscript abstract (include quotes in quotation marks "like this" to indicate direct quotes from your manuscript), or elaborate on this item by providing additional information not in the ms, or briefly explain why the item is not applicable/relevant for your study

First in the Methods it is provided like this "students recruited through multiple strategies consented and were randomized: WLC=40; F-MVC=40, P-MVC=39". Then, in the Results it is provided like this "Of the 113 students who provided T1 data.... Participants in F-MVC (n=39), P-MVC (n=35), and WLC (n=39) groups..."

1b-v) CONCLUSIONS/DISCUSSION in abstract for negative trials

Conclusions/Discussions in abstract for negative trials: Discuss the primary outcome - if the trial is negative (primary outcome not changed), and the intervention was not used, discuss whether negative results are attributable to lack of uptake and discuss reasons. (Note: Only report in the abstract what the main paper is reporting. If this information is missing from the main body of text, consider adding it)

	1	2	3	4	5	
subitem not at all important	○	○	○	◉	○	essential

Does your paper address subitem 1b-v?

Copy and paste relevant sections from the manuscript abstract (include quotes in quotation marks "like this" to indicate direct quotes from your manuscript), or elaborate on this item by providing additional information not in the ms, or briefly explain why the item is not applicable/relevant for your study

Not applicable as the intervention was effective for changing primary outcomes.

INTRODUCTION

2a) In INTRODUCTION: Scientific background and explanation of rationale

2a-i) Problem and the type of system/solution

Describe the problem and the type of system/solution that is object of the study: intended as stand-alone intervention vs. incorporated in broader health care program? Intended for a particular patient population? Goals of the intervention, e.g., being more cost-effective to other interventions, replace or complement other solutions? (Note: Details about the intervention are provided in "Methods" under 5)

	1	2	3	4	5	
subitem not at all important	○	○	○	○	◉	essential

Does your paper address subitem 2a-i? *

Copy and paste relevant sections from the manuscript (include quotes in quotation marks "like this" to indicate direct quotes from your manuscript), or elaborate on this item by providing additional information not in the ms, or briefly explain why the item is not applicable/relevant for your study

The information is provided like this "Mental health disorders, especially those involving depression, anxiety, and stress, are a rising problem among college students internationally. In the United States, analyses of college data show that mental health disorders are among the top five diagnostic categories seen at college health services and responsible for the highest number of visits per student (4.93) with depression and anxiety at the top [1]. Furthermore, multiple studies indicate an increasing prevalence of mental health disorders, especially depression and anxiety, in undergraduate students [1-8]. In Canada, a large study of nursing students indicated the prevalence of mild-to-severe depression, anxiety, and stress at 33%, 39%, and 38%, respectively [9]. Similar rates of mental health difficulties are reported among students from other countries [10-14]. The counseling centers in colleges and universities provide care to students in distress through various models such as clinical services, advising, awareness workshops, and training programs [15]. However, students often experience difficulties in accessing these services (eg, stigma and time concerns for in-person sessions along with financial cost for some services) [16,17], while counseling centers are overwhelmed due to limited resources. An analysis of Canadian colleges and universities revealed that enrollment in the province of Ontario increased by 27% between 2004 and 2012, but the budget for counseling centers increased by 5%, leading to just 1 campus-based counselor for 1300 to 4835 students [18]. Similarly, in the United States, a 2014 study indicated that the average ratio of counselors to students was 1 to 2081 [19]. New and accessible strategies are needed to address the students' mental health and at an early stage. One such approach is mindfulness-based techniques.

Mindfulness is defined as "the awareness that emerges through paying attention on purpose, in the present, and nonjudgmentally to the unfolding of experience moment by moment" [20]. The techniques learned in mindfulness practices involve nonjudgmental attention directed to each present moment. Although mindfulness meditation has been practiced for centuries in Buddhist and other spiritual traditions, its application to psychological health in the West emerged in 1980s when Jon Kabat-Zinn examined its clinical use in treating chronic pain [21]. This technique known as mindfulness-based stress reduction has a core focus on "intensive [and repeated] training of mindfulness meditation to help individuals relate to their physical and psychological conditions in a more accepting and nonjudgmental ways" [22]. Further scholarly work, such as by Segal et al [23], combined the principles of mindfulness with cognitive behavioral therapy (CBT). This program called mindfulness-based cognitive therapy (MBCT) has been researched for treating mental health conditions, especially depression. In addition to the principles of mindfulness practice, MBCT "aims to change one's awareness of and relationship to thoughts and emotions" to reduce the associations between negative automatic thinking and dysphoria [22]. Other psychotherapeutic techniques with mindfulness-orientation include dialectical behavior therapy and acceptance and commitment therapy, but the meditation practice is only one aspect of the full approach. Evidence shows that mindfulness-based interventions positively impact psychological [22,24] and physical health [25], with multiple meta-

2a-ii) Scientific background, rationale: What is known about the (type of) system

Scientific background, rationale: What is known about the (type of) system that is the object of the study (be sure to discuss the use of similar systems for other conditions/diagnoses, if appropiate), motivation for the study, i.e. what are the reasons for and what is the context for this specific study, from which stakeholder viewpoint is the study performed, potential impact of findings [2]. Briefly justify the choice of the comparator.

	1	2	3	4	5	
subitem not at all important	○	○	○	●	○	essential

Does your paper address subitem 2a-ii? *

Copy and paste relevant sections from the manuscript (include quotes in quotation marks "like this" to indicate direct quotes from your manuscript), or elaborate on this item by providing additional information not in the ms, or briefly explain why the item is not applicable/relevant for your study

Like this " In relation to student population, several recent reviews have indicated that in-person mindfulness-based interventions have a positive effect on students' mood and their levels of stress, anxiety, and depression [39-42].

However, a handful of student studies exist on Web-based mindfulness-based programs despite its potential to complement overstretched traditional counseling services on campuses [43]. This emerging scholarly work with students has examined the impact of Web-based mindfulness on a variety of mental health –related issues and demonstrated improvements in outcomes such as mental health, well-being, mindfulness, stress and depression symptoms, life satisfaction, and social connectedness [43-50]. However, the effectiveness of a Web-based mindfulness intervention when combined with the constructs of CBT remains an area requiring more rigorous examination. This is a missed opportunity given that systematic reviews show that internet-based CBT is significantly effective compared with control groups in reducing anxiety, especially when supported by therapist's email or phone contact [51], and in reducing depression symptoms [52]. There is the potential for substantial gains by combining these two techniques—mindfulness and CBT—through Web-based interventions for students who are also technologically fluent and capable; studies also indicate that students prefer to self-initiate help-seeking for Web-based services compared with in-person services [53]. There is also a need to better understand the optimal duration and delivery style of Web-based mindfulness-CBT interventions. Although durations of 6 to 8 weeks are more common, 2-week interventions [48] and a single Web session [47] have also been used. In terms of delivery, some of these studies supported the interventions with reminders, written feedback, and coaching, whereas others were passive. High attrition rate was a common problem in several student studies [44,45,48,50], although it was a significantly less prevalent problem in studies that used coaching, reminders, and feedback strategies [43,49]. Indeed, further scholarly work is needed to inform development of student-friendly and effective Web-based mindfulness-CBT programs. Thus, our team developed a Mindfulness Virtual Community (MVC) Web-based program (described below) after conducting eight

2b) In INTRODUCTION: Specific objectives or hypotheses

Does your paper address CONSORT subitem 2b? *

Copy and paste relevant sections from the manuscript (include quotes in quotation marks "like this" to indicate direct quotes from your manuscript), or elaborate on this item by providing additional information not in the ms, or briefly explain why the item is not applicable/relevant for your study

Like this ""Thus, our team developed a Mindfulness Virtual Community (MVC) Web-based program (described below) after conducting eight focus groups with students and incorporating comprehensive review of pertinent literature [54-58].

Study Objective
To examine the efficacy of an MVC program for mental health among undergraduate students in a Canadian university, we conducted a pilot RCT with 4 parallel arms: full MVC (F-MVC), partial MVC (P-MVC), waitlist control (WLC), and group-based face-to-face CBT mindfulness. As the main focus of the trial was to examine the MVC program, we report here the impact of F-MVC and P-MVC vs WLC; the results for face-to-face CBT mindfulness are presented elsewhere. The primary outcomes were symptoms of depression, anxiety, and stress, and secondary outcomes were quality of life, life satisfaction, and mindfulness. It was hypothesized that (1) symptom scores for depression, anxiety, and stress at 8 weeks (T3) will be significantly improved in the F-MVC group when compared with the WLC group and (2) scores for quality of life, life satisfaction, and mindfulness at T3 will be significantly better for the F-MVC intervention group than the WLC group. The P-MVC intervention was included to explore a significantly less expensive alternative to delivering beneficial effects and was hypothesized to have similar but

METHODS

3a) Description of trial design (such as parallel, factorial) including allocation ratio

Does your paper address CONSORT subitem 3a? *

Copy and paste relevant sections from the manuscript (include quotes in quotation marks "like this" to indicate direct quotes from your manuscript), or elaborate on this item by providing additional information not in the ms, or briefly explain why the item is not applicable/relevant for your study

Like this "These students started the parallel-arm RCT on January 16, 2017", and like this "Participating students were randomized to either the F-MVC intervention, P-MVC intervention, WLC, or a face-to-face CBT mindfulness group using 1:1:1:1 block randomization. We report here the impact of F-MVC and P-MVC vs WLC, whereas the results for face-to-face CBT mindfulness are being presented elsewhere (manuscript under review). The randomization allocation sequence was computer-generated by an off-site team member who concealed it in sequentially numbered, opaque envelopes [61]. These envelopes were opened only after a written consent, keeping participants and research assistants blind to allocation."

3b) Important changes to methods after trial commencement (such as eligibility criteria), with reasons

Does your paper address CONSORT subitem 3b? *

Copy and paste relevant sections from the manuscript (include quotes in quotation marks "like this" to indicate direct quotes from your manuscript), or elaborate on this item by providing additional information not in the ms, or briefly explain why the item is not applicable/relevant for your study

There were no changes in the eligibility criteria or recruitment methods.

3b-i) Bug fixes, Downtimes, Content Changes

Bug fixes, Downtimes, Content Changes: ehealth systems are often dynamic systems. A description of changes to methods therefore also includes important changes made on the intervention or comparator during the trial (e.g., major bug fixes or changes in the functionality or content) (5-iii) and other “unexpected events” that may have influenced study design such as staff changes, system failures/downtimes, etc. [2].

	1	2	3	4	5	
subitem not at all important	○	○	○	◉	○	essential

Does your paper address subitem 3b-i?

Copy and paste relevant sections from the manuscript (include quotes in quotation marks "like this" to indicate direct quotes from your manuscript), or elaborate on this item by providing additional information not in the ms, or briefly explain why the item is not applicable/relevant for your study

The main paper also provides information about the intervention like this "Once the intervention was deployed, the content of modules and platform structure remained unchanged."

4a) Eligibility criteria for participants

Does your paper address CONSORT subitem 4a? *

Copy and paste relevant sections from the manuscript (include quotes in quotation marks "like this" to indicate direct quotes from your manuscript), or elaborate on this item by providing additional information not in the ms, or briefly explain why the item is not applicable/relevant for your study

"Student eligibility criteria were a minimal age of 18 years, English language fluency, self-reported high level of confidence to complete the study, and current undergraduate student status. Their ability to use a computer and smartphone and internet literacy were assumed to be de facto skills. The study was advertised as "Mindfulness Approaches to Wellbeing on Campus" and used multiple recruitment strategies including study posters, class visits on permission of course directors, and email invitations via listservs of student associations in the Faculty of Health and Faculty of Liberal Arts. Interested students contacted the research staff via email or phone and were further screened for substance abuse and indications of psychoses (ie, hallucinations). If either of these two conditions "interfered in routine life within last month," they were excluded and provided a list of mental health resources for access. Eligible and willing students received detailed information in-person about the study and provided informed

4a-i) Computer / Internet literacy

Computer / Internet literacy is often an implicit "de facto" eligibility criterion - this should be explicitly clarified.

	1	2	3	4	5	
subitem not at all important	○	○	○	●	○	essential

Does your paper address subitem 4a-i?

Copy and paste relevant sections from the manuscript (include quotes in quotation marks "like this" to indicate direct quotes from your manuscript), or elaborate on this item by providing additional information not in the ms, or briefly explain why the item is not applicable/relevant for your study

Like this "Their ability to use a computer and smartphone and internet literacy were assumed to be de facto skills."

4a-ii) Open vs. closed, web-based vs. face-to-face assessments:

Open vs. closed, web-based vs. face-to-face assessments: Mention how participants were recruited (online vs. offline), e.g., from an open access website or from a clinic, and clarify if this was a purely web-based trial, or there were face-to-face components (as part of the intervention or for assessment), i.e., to what degree got the study team to know the participant. In online-only trials, clarify if participants were quasi-anonymous and whether having multiple identities was possible or whether technical or logistical measures (e.g., cookies, email confirmation, phone calls) were used to detect/prevent these.

	1	2	3	4	5	
subitem not at all important	○	○	○	○	●	essential

Does your paper address subitem 4a-ii? *

Copy and paste relevant sections from the manuscript (include quotes in quotation marks "like this" to indicate direct quotes from your manuscript), or elaborate on this item by providing additional information not in the ms, or briefly explain why the item is not applicable/relevant for your study

Like this in the Recruitment and Randomization section "The study was advertised as “Mindfulness Approaches to Wellbeing on Campus” and used multiple recruitment strategies including study posters, class visits on permission of course directors, and email invitations via listservs of student associations in the Faculty of Health and Faculty of Liberal Arts. Interested students contacted the research staff via email or phone and were further screened for substance abuse and indications of psychoses (ie, hallucinations). If either of these two conditions 'interfered in routine life within last month,' they were excluded and provided a list of mental health resources for access. Eligible and willing students received detailed information in-person about the study and provided informed written consent." In the same in-person meeting the research assistant opened the opaque envelopes with concealed allocations after written consent and students were randomized.

In the Recruitment and Randomization section, we also provide information on how we withheld the possibility of multiple-account creation by the participants like this "Each participant received a unique ID number. Those in the F-MVC and P-MVC groups also received a temporary password to access the Web-based intervention; they changed the password after first login while IDs remained the same to eliminate the possibility of creating multiple accounts or identities."

In the section on Study Arms, we provide more information on the contact with the research staff like this "The students in both [intervention] groups received email reminders from the project staff before the release of each new module, whereas the reminders for conference chat were sent only to the F-MVC group."

The measurement of demographics and outcomes were all online and its described

4a-iii) Information giving during recruitment

Information given during recruitment. Specify how participants were briefed for recruitment and in the informed consent procedures (e.g., publish the informed consent documentation as appendix, see also item X26), as this information may have an effect on user self-selection, user expectation and may also bias results.

	1	2	3	4	5	
subitem not at all important	○	○	○	◉	○	essential

Does your paper address subitem 4a-iii?

Copy and paste relevant sections from the manuscript (include quotes in quotation marks "like this" to indicate direct quotes from your manuscript), or elaborate on this item by providing additional information not in the ms, or briefly explain why the item is not applicable/relevant for your study

The information is provided like this "Interested students contacted the research staff via email or phone and were further screened for substance abuse and indications of psychoses (ie, hallucinations). If either of these two conditions “interfered in routine life within last month,” they were excluded and provided a list of mental health resources for access. Eligible and willing students received detailed information in-person about the study and provided informed written consent. Participants were able to select an honorarium of Can $50 or 2% in course grade (for professors who gave this permission) or three credits (equivalent to 2% course grade) in the Undergraduate Research Participation Pool of the Department of Psychology. Each participant also received a resource list that included information about health and social services on campus and in the community (eg, 24×7 “Good To Talk” helpline for postsecondary students in Ontario). Although our study participants largely comprised healthy volunteers, our protocol included a safety mechanism whereby participants were asked verbally and in the consent form to contact the research staff if they felt distress during the trial period so that “limited counselling with a clinical psychologist could be arranged, if needed”; the collaborating psychologist was at arms’ length from the

4b) Settings and locations where the data were collected

Does your paper address CONSORT subitem 4b? *

Copy and paste relevant sections from the manuscript (include quotes in quotation marks "like this" to indicate direct quotes from your manuscript), or elaborate on this item by providing additional information not in the ms, or briefly explain why the item is not applicable/relevant for your study

The outcomes were measured by online surveys completed by students. This is described in the paper under the Measurement section like this "Participants in all groups completed online surveys at T1, T2, and T3."

4b-i) Report if outcomes were (self-)assessed through online questionnaires

Clearly report if outcomes were (self-)assessed through online questionnaires (as common in web-based trials) or otherwise.

	1	2	3	4	5	
subitem not at all important	○	○	○	◉	○	essential

Does your paper address subitem 4b-i? *

Copy and paste relevant sections from the manuscript (include quotes in quotation marks "like this" to indicate direct quotes from your manuscript), or elaborate on this item by providing additional information not in the ms, or briefly explain why the item is not applicable/relevant for your study

The outcomes were measured by online surveys completed by students. This is described in the paper under the Measurement section like this "Participants in all groups completed online surveys at T1, T2, and T3."

4b-ii) Report how institutional affiliations are displayed

Report how institutional affiliations are displayed to potential participants [on ehealth media], as affiliations with prestigious hospitals or universities may affect volunteer rates, use, and reactions with regards to an intervention.(Not a required item – describe only if this may bias results)

	1	2	3	4	5	
subitem not at all important	○	○	◉	○	○	essential

Does your paper address subitem 4b-ii?

Copy and paste relevant sections from the manuscript (include quotes in quotation marks "like this" to indicate direct quotes from your manuscript), or elaborate on this item by providing additional information not in the ms, or briefly explain why the item is not applicable/relevant for your study

Please see the screen shot in Figure 2 in the paper. Only the university name, which received the research grant, and the partnering IT company name appear on it.

5) The interventions for each group with sufficient details to allow replication, including how and when they were actually administered

5-i) Mention names, credential, affiliations of the developers, sponsors, and owners

Mention names, credential, affiliations of the developers, sponsors, and owners [6] (if authors/evaluators are owners or developer of the software, this needs to be declared in a “Conflict of interest” section or mentioned elsewhere in the manuscript).

	1	2	3	4	5	
subitem not at all important	○	○	○	◉	○	essential

Does your paper address subitem 5-i?

Copy and paste relevant sections from the manuscript (include quotes in quotation marks "like this" to indicate direct quotes from your manuscript), or elaborate on this item by providing additional information not in the ms, or briefly explain why the item is not applicable/relevant for your study

Under the section The Mindfulness Virtual Community Program, we provide information "The modules' topics were informed by our findings from the focus groups with students [54,55]. The module scripts and audio recordings were created by one of the investigator with extensive clinical experience (PR) and drew from combined mindfulness and CBT principles. The choice of moving and still images used in the creation of the videos involved collaborative work (PR, CE, and FA)."

In the same section, more information is provided "The Web platform for the intervention had separate logins for the student participant and the videoconference moderator (Figure 2). This was developed in partnership with the industry partner, ForaHealthyme Inc....The moderator (independent counselor with a master's degree in psychology and training in mindfulness) had weekly discussions with the team clinician (PR) to optimize engagement during videoconferences."

The Conflict of Interest section states "It is the understanding of the university and researchers that the Project Intellectual Property belongs to the CE, FA, and PR. The industry partner ForaHealthyme has title to the copyrights of any computer source code software that was developed out of this research project."

The Acknowledgement section statement includes "The work reported in this paper was funded by the Canadian Institutes for Health Research (CIHR), eHealth Innovations Partnership Program Grant; grant number EH1-143553. The project principal investigators are CE (nominated), FA, and PR."

Further, "The data analysis was conducted by biostatistician on our team (RM) who was not involved in the content development of the intervention and its deployment. The statistical software SAS 9.4 was used for statistical analyses."

5-ii) Describe the history/development process

Describe the history/development process of the application and previous formative evaluations (e.g., focus groups, usability testing), as these will have an impact on adoption/use rates and help with interpreting results.

	1	2	3	4	5	
subitem not at all important	○	○	○	◉	○	essential

Does your paper address subitem 5-ii?

Copy and paste relevant sections from the manuscript (include quotes in quotation marks "like this" to indicate direct quotes from your manuscript), or elaborate on this item by providing additional information not in the ms, or briefly explain why the item is not applicable/relevant for your study

We describe it like this "Each of the 12 modules consists of one educational content and one mindfulness-practice content video, recorded in male and female voices with low volume background music, and offered in high- and low-resolution videos (a total of 8 videos per module). The modules' topics were informed by our findings from the focus groups with students [54,55]. The module scripts and audio recordings were created by one of the investigators with extensive clinical experience (PR) and drew from combined mindfulness and CBT principles. The choice of moving and still images used in the creation of the videos involved collaborative work (PR, CE, and FA).

The Web platform for the intervention had separate logins for the student participant and the videoconference moderator (Figure 2). This was developed in partnership with the industry partner, ForaHealthyme Inc.......The moderator (independent counselor with a master's degree in psychology and training in mindfulness) had weekly discussions with the team clinician (PR) to optimize engagement during videoconferences. Once the intervention was deployed, the content of modules and platform structure remained unchanged."

5-iii) Revisions and updating

Revisions and updating. Clearly mention the date and/or version number of the application/intervention (and comparator, if applicable) evaluated, or describe whether the intervention underwent major changes during the evaluation process, or whether the development and/or content was “frozen” during the trial. Describe dynamic components such as news feeds or changing content which may have an impact on the replicability of the intervention (for unexpected events see item 3b).

	1	2	3	4	5	
subitem not at all important	○	○	○	●	○	essential

Does your paper address subitem 5-iii?

Copy and paste relevant sections from the manuscript (include quotes in quotation marks "like this" to indicate direct quotes from your manuscript), or elaborate on this item by providing additional information not in the ms, or briefly explain why the item is not applicable/relevant for your study

its provided like this "Once the intervention was deployed, the content of modules and platform structure remained unchanged."

5-iv) Quality assurance methods

Provide information on quality assurance methods to ensure accuracy and quality of information provided [1], if applicable.

	1	2	3	4	5	
subitem not at all important	○	○	○	○	●	essential

Does your paper address subitem 5-iv?

Copy and paste relevant sections from the manuscript (include quotes in quotation marks "like this" to indicate direct quotes from your manuscript), or elaborate on this item by providing additional information not in the ms, or briefly explain why the item is not applicable/relevant for your study

Summary of some of the quality elements: We used the parallel-arm RCT design and registered it before collecting data and starting the intervention delivery. We provide comprehensive details about the development of intervention and conducted focus groups to inform its content and design. We used validated scales to measure the primary and secondary outcomes. The group allocation was generated by an off-site biostatistician and it was sealed in opaque envelopes. The participants and research assistant were blind to the allocation until after the written consent.

In the paper, an example of ensuring the quality process in the delivery/use/measurement of intervention is "Each participant received a unique ID number. Those in the F-MVC and P-MVC groups also received a temporary password to access the Web-based intervention; they changed the password after first login while IDs remained the same to eliminate the possibility of creating multiple accounts or identities."

The statistical analysis section provides several details, such as :
1. "The data analysis was conducted by biostatistician on our team (RM) who was not involved in the content development of the intervention and its deployment. The statistical software SAS 9.4 was used for statistical analyses."

2. "To account for multiple comparisons, due to the multiple outcomes analyzed, we considered P<.02 as statistically significant."

3. "The approach to the outcome analysis was Intention-to-Treat. First, we analyzed the data without any imputation for missing values and then repeated the analysis with an imputation of missing values using a last observation carried forward (LOCF) method. The results were similar for the complete-case analysis and analysis with LOCF; both are reported (see Multimedia Appendices 1 and 2)."

5-v) Ensure replicability by publishing the source code, and/or providing screenshots/screen-capture video, and/or providing flowcharts of the algorithms used

Ensure replicability by publishing the source code, and/or providing screenshots/screen-capture video, and/or providing flowcharts of the algorithms used. Replicability (i.e., other researchers should in principle be able to replicate the study) is a hallmark of scientific reporting.

	1	2	3	4	5	
subitem not at all important	○	○	○	◉	○	essential

Does your paper address subitem 5-v?

Copy and paste relevant sections from the manuscript (include quotes in quotation marks "like this" to indicate direct quotes from your manuscript), or elaborate on this item by providing additional information not in the ms, or briefly explain why the item is not applicable/relevant for your study

Figure 1 presents the components of the full intervention. The details on the module content are provided in Table 1 for the 12 topics and duration of each. Textbox1 provides examples of 2 modules for the psychoeducation part and the mindfulness practice part. A screen shot provides information on the look of the platform for users. A flow chart provides the recruitment process.

teh5-vi) Digital preservation

Digital preservation: Provide the URL of the application, but as the intervention is likely to change or disappear over the course of the years; also make sure the intervention is archived (Internet Archive, webcitation.org, and/or publishing the source code or screenshots/videos alongside the article). As pages behind login screens cannot be archived, consider creating demo pages which are accessible without login.

	1	2	3	4	5	
subitem not at all important	○	○	◉	○	○	essential

Does your paper address subitem 5-vi?

Copy and paste relevant sections from the manuscript (include quotes in quotation marks "like this" to indicate direct quotes from your manuscript), or elaborate on this item by providing additional information not in the ms, or briefly explain why the item is not applicable/relevant for your study

Screen shot is provided in the paper.

5-vii) Access

Access: Describe how participants accessed the application, in what setting/context, if they had to pay (or were paid) or not, whether they had to be a member of specific group. If known, describe how participants obtained “access to the platform and Internet” [1]. To ensure access for editors/reviewers/readers, consider to provide a “backdoor” login account or demo mode for reviewers/readers to explore the application (also important for archiving purposes, see vi).

	1	2	3	4	5	
subitem not at all important	○	○	○	○	○	essential

Does your paper address subitem 5-vii? *

Copy and paste relevant sections from the manuscript (include quotes in quotation marks "like this" to indicate direct quotes from your manuscript), or elaborate on this item by providing additional information not in the ms, or briefly explain why the item is not applicable/relevant for your study

The participation was completely voluntary and its described in the paper as “Student eligibility criteria were a minimal age of 18 years, English language fluency, self-reported high level of confidence to complete the study, and current undergraduate student status. Their ability to use a computer and smartphone and internet literacy were assumed to be de facto skills. The study was advertised as “Mindfulness Approaches to Wellbeing on Campus” and used multiple recruitment strategies including study posters, class visits on permission of course directors, and email invitations via listservs of student associations in the Faculty of Health and Faculty of Liberal Arts. Interested students contacted the research staff via email or phone and were further screened for substance abuse and indications of psychoses (ie, hallucinations). If either of these two conditions “interfered in routine life within last month,” they were excluded and provided a list of mental health resources for access. Eligible and willing students received detailed information in-person about the study and provided informed written consent. Participants were able to select an honorarium of Can $50 or 2% in course grade (for professors who gave this permission) or three credits (equivalent to 2% course grade) in the Undergraduate Research Participation Pool of the Department of Psychology. Each participant also received a resource list that included information about health and social services on campus and in the community (eg, 24×7 “Good To Talk” helpline for postsecondary students in Ontario). Although our study participants largely comprised healthy volunteers, our protocol included a safety mechanism whereby participants were asked verbally and in the consent form to contact the research staff if they felt distress during the

5-viii) Mode of delivery, features/functionalities/components of the intervention and comparator, and the theoretical framework

Describe mode of delivery, features/functionalities/components of the intervention and comparator, and the theoretical framework [6] used to design them (instructional strategy [1], behaviour change techniques, persuasive features, etc., see e.g., [7, 8] for terminology). This includes an in-depth description of the content (including where it is coming from and who developed it) [1],” whether [and how] it is tailored to individual circumstances and allows users to track their progress and receive feedback” [6]. This also includes a description of communication delivery channels and – if computer-mediated communication is a component – whether communication was synchronous or asynchronous [6]. It also includes information on presentation strategies [1], including page design principles, average amount of text on pages, presence of hyperlinks to other resources, etc. [1].

	1	2	3	4	5	
subitem not at all important	○	○	○	○	○	essential

Does your paper address subitem 5-viii? *

Copy and paste relevant sections from the manuscript (include quotes in quotation marks "like this" to indicate direct quotes from your manuscript), or elaborate on this item by providing additional information not in the ms, or briefly explain why the item is not applicable/relevant for your study

“The Mindfulness Virtual Community Program:
A total of three components of the Web-based MVC program (Figure 1) were (1) youth-specific mental health education and mindfulness-practice modules, delivered via video recordings for participants to watch and listen to on personal computers, phones, and tablets at convenient times; (2) anonymous, asynchronous peer-to-peer discussion boards pertaining to mental health and mindfulness practice; and (3) anonymous, 20-min live videoconferences (group-based) on module topics guided by a mental health professional.

Each of the 12 modules consists of one educational content and one mindfulness-practice content video, recorded in male and female voices with low volume background music, and offered in high- and low-resolution videos (a total of 8 videos per module). The modules’ topics were informed by our findings from the focus groups with students [54,55]. The module scripts and audio recordings were created by one of the investigators with extensive clinical experience (PR) and drew from combined mindfulness and CBT principles. The choice of moving and still images used in the creation of the videos involved collaborative work (PR, CE, and FA). Table 1 lists the topics of 12 modules and video duration (average of male and female voice), and Textbox 1 provides examples of the module content.

The Web platform for the intervention had separate logins for the student participant and the videoconference moderator (Figure 2). This was developed in partnership with the industry partner, ForaHealthyme Inc. The F-MVC student version provided access to the video-based modules, text-based peer-to-peer discussion forum, a calendar to book an upcoming live videoconference, a video room (camera being off as default) with ability to privately text the moderator, and a resource page with contact information on various social and health services. The moderator version offered access to the student version along with additional features such as populating the calendar with dates and times for the live videoconferences, starting the videoconference with camera turned on for the moderator by default, and responding privately to incoming text messages. The moderator (independent counselor with a master’s degree in psychology and training in mindfulness) had weekly discussions with the team clinician (PR) to optimize engagement during videoconferences. Once the intervention was

5-ix) Describe use parameters

Describe use parameters (e.g., intended “doses” and optimal timing for use). Clarify what instructions or recommendations were given to the user, e.g., regarding timing, frequency, heaviness of use, if any, or was the intervention used ad libitum.

	1	2	3	4	5	
subitem not at all important	○	○	○	◉	○	essential

Does your paper address subitem 5-ix?

Copy and paste relevant sections from the manuscript (include quotes in quotation marks "like this" to indicate direct quotes from your manuscript), or elaborate on this item by providing additional information not in the ms, or briefly explain why the item is not applicable/relevant for your study

"The partial and full intervention participants were instructed to use the platform ad

5-x) Clarify the level of human involvement

Clarify the level of human involvement (care providers or health professionals, also technical assistance) in the e-intervention or as co-intervention (detail number and expertise of professionals involved, if any, as well as “type of assistance offered, the timing and frequency of the support, how it is initiated, and the medium by which the assistance is delivered”. It may be necessary to distinguish between the level of human involvement required for the trial, and the level of human involvement required for a routine application outside of a RCT setting (discuss under item 21 – generalizability).

	1	2	3	4	5	
subitem not at all important	○	○	○	○	○	essential

Does your paper address subitem 5-x?

Copy and paste relevant sections from the manuscript (include quotes in quotation marks "like this" to indicate direct quotes from your manuscript), or elaborate on this item by providing additional information not in the ms, or briefly explain why the item is not applicable/relevant for your study

Your answer

5-xi) Report any prompts/reminders used

Report any prompts/reminders used: Clarify if there were prompts (letters, emails, phone calls, SMS) to use the application, what triggered them, frequency etc. It may be necessary to distinguish between the level of prompts/reminders required for the trial, and the level of prompts/reminders for a routine application outside of a RCT setting (discuss under item 21 – generalizability).

	1	2	3	4	5	
subitem not at all important	○	○	○	◉	○	essential

Does your paper address subitem 5-xi? *

Copy and paste relevant sections from the manuscript (include quotes in quotation marks "like this" to indicate direct quotes from your manuscript), or elaborate on this item by providing additional information not in the ms, or briefly explain why the item is not applicable/relevant for your study

"The students in both groups received email reminders from the project staff before the release of each new module, whereas the reminders for conference chat were sent only to the F-MVC group."

5-xii) Describe any co-interventions (incl. training/support)

Describe any co-interventions (incl. training/support): Clearly state any interventions that are provided in addition to the targeted eHealth intervention, as ehealth intervention may not be designed as stand-alone intervention. This includes training sessions and support [1]. It may be necessary to distinguish between the level of training required for the trial, and the level of training for a routine application outside of a RCT setting (discuss under item 21 – generalizability.

	1	2	3	4	5	
subitem not at all important	○	○	○	◉	○	essential

Does your paper address subitem 5-xii? *

Copy and paste relevant sections from the manuscript (include quotes in quotation marks "like this" to indicate direct quotes from your manuscript), or elaborate on this item by providing additional information not in the ms, or briefly explain why the item is not applicable/relevant for your study

No other co-intervention was provided.

6a) Completely defined pre-specified primary and secondary outcome measures, including how and when they were assessed

Does your paper address CONSORT subitem 6a? *

Copy and paste relevant sections from the manuscript (include quotes in quotation marks "like this" to indicate direct quotes from your manuscript), or elaborate on this item by providing additional information not in the ms, or briefly explain why the item is not applicable/relevant for your study

"Main Outcomes and Measurement:
Participants in all groups completed online surveys at T1, T2, and T3. The primary outcomes were depression, anxiety, and stress symptoms. For the measurement of depression symptoms, we used the 9-item Patient Health Questionnaire (PHQ-9) [62]; each item is rated on a scale of 0 to 3, and the total score range is 0 to 27 (score 0-9 indicates no/subclinical level of depression, 10-14 moderate, 15-19 moderately severe, and ≥20 severe). The symptoms of anxiety were measured by using the 21-item Beck Anxiety Inventory (BAI) [63]; each item is rated on a scale of 0 to 3, and the total score range is 0 to 63 (score 0-21 indicates no/low level of anxiety, 22-35 moderate, and ≥36 severe). For the measurement of stress, we used the 10-item Perceived Stress Scale (PSS) [64]; each item is rated on a scale of 0 to 4, and the total score range is 0 to 40 (score 0-13 indicates mild level of stress, 14-26 moderate, and 27-40 high). The secondary outcomes were quality of life, life satisfaction, and mindfulness. We used the 16-item Quality of Life Scale (QOLS) [65], which has a total score range of 16 to 112, and each item is rated on a scale of 1 to 7. The student life satisfaction was measured by using the 6-item Brief Multidimensional Students' Life Satisfaction Scale-Peabody Treatment Progress Battery (BMSLSS-PTPB) [66]; each item is rated on a scale of 1 to 5, and item scores are averaged together to give a total score that ranges from 1 to 5. The level of mindfulness was measured by the 24-item Five-Facet Mindfulness Questionnaire-Short Form (FFMQ-SF) [67]; each item is rated on a scale of 1 to 5, and the total score range is 24 to 120. The subscales in the FFMQ-SF are nonreactivity to inner experience (5 items), observing (4 items), acting with awareness (5 items), describing (5 items), and nonjudging of inner experience (5 items). We assessed each of the scale for internal consistency at the T1, T2, and T3 datasets, and Cronbach alpha ranged from .82 to .94: PHQ-9 .87, .89, and .86; BAI .93, .93, and .94; PSS .90, .89, and .90; QOLS .87, .91, and .92; BMSLSS-PTPB .82, .85,

6a-i) Online questionnaires: describe if they were validated for online use and apply CHERRIES items to describe how the questionnaires were designed/deployed

If outcomes were obtained through online questionnaires, describe if they were validated for online use and apply CHERRIES items to describe how the questionnaires were designed/deployed [9].

	1	2	3	4	5	
subitem not at all important	○	○	○	○	○	essential

Does your paper address subitem 6a-i?

Copy and paste relevant sections from manuscript text

Your answer

6a-ii) Describe whether and how “use” (including intensity of use/dosage) was defined/measured/monitored

Describe whether and how “use” (including intensity of use/dosage) was defined/measured/monitored (logins, logfile analysis, etc.). Use/adoption metrics are important process outcomes that should be reported in any ehealth trial.

	1	2	3	4	5	
subitem not at all important	○	○	○	○	○	essential

Does your paper address subitem 6a-ii?

Copy and paste relevant sections from manuscript text

Your answer

6a-iii) Describe whether, how, and when qualitative feedback from participants was obtained

Describe whether, how, and when qualitative feedback from participants was obtained (e.g., through emails, feedback forms, interviews, focus groups).

	1	2	3	4	5	
subitem not at all important	○	○	○	○	○	essential

Does your paper address subitem 6a-iii?

Copy and paste relevant sections from manuscript text

Your answer

6b) Any changes to trial outcomes after the trial commenced, with reasons

Does your paper address CONSORT subitem 6b? *

Copy and paste relevant sections from the manuscript (include quotes in quotation marks "like this" to indicate direct quotes from your manuscript), or elaborate on this item by providing additional information not in the ms, or briefly explain why the item is not applicable/relevant for your study

No changes were made.

7a) How sample size was determined

NPT: When applicable, details of whether and how the clustering by care provides or centers was addressed

7a-i) Describe whether and how expected attrition was taken into account when calculating the sample size

Describe whether and how expected attrition was taken into account when calculating the sample size.

	1	2	3	4	5	
subitem not at all important	○	○	◉	○	○	essential

Does your paper address subitem 7a-i?

Copy and paste relevant sections from manuscript title (include quotes in quotation marks "like this" to indicate direct quotes from your manuscript), or elaborate on this item by providing additional information not in the ms, or briefly explain why the item is not applicable/relevant for your study

Your answer

7b) When applicable, explanation of any interim analyses and stopping guidelines

Does your paper address CONSORT subitem 7b? *

Copy and paste relevant sections from the manuscript (include quotes in quotation marks "like this" to indicate direct quotes from your manuscript), or elaborate on this item by providing additional information not in the ms, or briefly explain why the item is not applicable/relevant for your study

"Although our study participants largely comprised healthy volunteers, our protocol included a safety mechanism whereby participants were asked verbally and in the consent form to contact the research staff if they felt distress during the trial period so that “limited counselling with a clinical psychologist could be arranged, if needed”; the collaborating psychologist was at arms’ length from the trial. No instance of such request arose during the reported study."

There was no interim analysis planned given the pilot nature of the study.

8a) Method used to generate the random allocation sequence

NPT: When applicable, how care providers were allocated to each trial group

Does your paper address CONSORT subitem 8a? *

Copy and paste relevant sections from the manuscript (include quotes in quotation marks "like this" to indicate direct quotes from your manuscript), or elaborate on this item by providing additional information not in the ms, or briefly explain why the item is not applicable/relevant for your study

"The randomization allocation sequence was computer-generated by an off-site team member who concealed it in sequentially numbered, opaque envelopes [61]. These envelopes were opened only after a written consent, keeping participants and research assistants blind to allocation."

8b) Type of randomisation; details of any restriction (such as blocking and block size)

Does your paper address CONSORT subitem 8b? *

Copy and paste relevant sections from the manuscript (include quotes in quotation marks "like this" to indicate direct quotes from your manuscript), or elaborate on this item by providing additional information not in the ms, or briefly explain why the item is not applicable/relevant for your study

"Participating students were randomized to either the F-MVC intervention, P-MVC intervention, WLC, or a face-to-face CBT mindfulness group using 1:1:1:1 block randomization. We report here the impact of F-MVC and P-MVC vs WLC, whereas the results for face-to-face CBT mindfulness are being presented elsewhere (manuscript under review). "

9) Mechanism used to implement the random allocation sequence (such as sequentially numbered containers), describing any steps taken to conceal the sequence until interventions were assigned

Does your paper address CONSORT subitem 9? *

Copy and paste relevant sections from the manuscript (include quotes in quotation marks "like this" to indicate direct quotes from your manuscript), or elaborate on this item by providing additional information not in the ms, or briefly explain why the item is not applicable/relevant for your study

"The randomization allocation sequence was computer-generated by an off-site team member who concealed it in sequentially numbered, opaque envelopes [61]. These envelopes were opened only after a written consent, keeping participants and research assistants blind to allocation."

10) Who generated the random allocation sequence, who enrolled participants, and who assigned participants to interventions

Does your paper address CONSORT subitem 10? *

Copy and paste relevant sections from the manuscript (include quotes in quotation marks "like this" to indicate direct quotes from your manuscript), or elaborate on this item by providing additional information not in the ms, or briefly explain why the item is not applicable/relevant for your study

"The randomization allocation sequence was computer-generated by an off-site team member who concealed it in sequentially numbered, opaque envelopes [61]. These envelopes were opened only after a written consent, keeping participants and research assistants blind to allocation."

11a) If done, who was blinded after assignment to interventions (for example, participants, care providers, those assessing outcomes) and how

NPT: Whether or not administering co-interventions were blinded to group assignment

11a-i) Specify who was blinded, and who wasn’t

Specify who was blinded, and who wasn’t. Usually, in web-based trials it is not possible to blind the participants [1, 3] (this should be clearly acknowledged), but it may be possible to blind outcome assessors, those doing data analysis or those administering co-interventions (if any).

	1	2	3	4	5	
subitem not at all important	○	○	○	○	○	essential

Does your paper address subitem 11a-i? *

Copy and paste relevant sections from the manuscript (include quotes in quotation marks "like this" to indicate direct quotes from your manuscript), or elaborate on this item by providing additional information not in the ms, or briefly explain why the item is not applicable/relevant for your study

"The randomization allocation sequence was computer-generated by an off-site team member who concealed it in sequentially numbered, opaque envelopes [61]. These envelopes were opened only after a written consent, keeping participants and research assistants blind to allocation."

11a-ii) Discuss e.g., whether participants knew which intervention was the "intervention of interest" and which one was the "comparator"

Informed consent procedures (4a-ii) can create biases and certain expectations - discuss e.g., whether participants knew which intervention was the "intervention of interest" and which one was the "comparator".

	1	2	3	4	5	
subitem not at all important	○	○	○	○	○	essential

Does your paper address subitem 11a-ii?

Copy and paste relevant sections from the manuscript (include quotes in quotation marks "like this" to indicate direct quotes from your manuscript), or elaborate on this item by providing additional information not in the ms, or briefly explain why the item is not applicable/relevant for your study

Your answer

11b) If relevant, description of the similarity of interventions

(this item is usually not relevant for ehealth trials as it refers to similarity of a placebo or sham intervention to a active medication/intervention)

Does your paper address CONSORT subitem 11b? *

Copy and paste relevant sections from the manuscript (include quotes in quotation marks "like this" to indicate direct quotes from your manuscript), or elaborate on this item by providing additional information not in the ms, or briefly explain why the item is not applicable/relevant for your study

Not applicable

12a) Statistical methods used to compare groups for primary and secondary outcomes

NPT: When applicable, details of whether and how the clustering by care providers or centers was addressed

Does your paper address CONSORT subitem 12a? *

Copy and paste relevant sections from the manuscript (include quotes in quotation marks "like this" to indicate direct quotes from your manuscript), or elaborate on this item by providing additional information not in the ms, or briefly explain why the item is not applicable/relevant for your study

“The trial data were first analyzed using descriptive statistics (means, frequencies, and proportions) to describe the sample characteristics for the control, partial intervention, and the full intervention groups at T1. The mean scores for each of the 6 scales (ie, primary and secondary outcomes) were calculated for the T1, T2, and T3 for the three groups. Effect size was calculated using Cohen d, by subtracting the mean of the treatment group from the mean of the control group and by dividing the mean difference with the pooled SD.

The approach to the outcome analysis was Intention-to-Treat. First, we analyzed the data without any imputation for missing values and then repeated the analysis with an imputation of missing values using a last observation carried forward (LOCF) method. The results were similar for the complete-case analysis and analysis with LOCF; both are reported (see Multimedia Appendices 1 and 2). The attrition rates across the three groups were low and similar between T1 and T3 (2 for F-MVC, 1 for P-MVC, and 1 for WLC). To compare score changes over time for the outcomes, linear regression analysis was done. The generalized estimation equation (GEE) with AR(1) covariance structure was used to adjust for repeated measures. The result of GEE analysis has the interpretation of population average. The mean score differences were calculated between groups and adjusted for potential confounding variables (ie, gender, age, country of birth, paid work, unpaid work, vigorous physical activities, self-rated health, and access to mental health private counseling via insurance). This choice of confounding variables was based on existing knowledge and theory. Scales were calculated as follows: if the number of missing items was more than half of the number of items of a scale, the scale was considered missing; otherwise the missing items were imputed in accord with the average of the nonmissing items of the scale. To account for multiple comparisons, due to the multiple outcomes analyzed, we considered $P<.02$ as statistically significant. The data analysis was conducted by biostatistician on our team (RM) who was not involved in the content development of the intervention and

12a-i) Imputation techniques to deal with attrition / missing values

Imputation techniques to deal with attrition / missing values: Not all participants will use the intervention/comparator as intended and attrition is typically high in ehealth trials. Specify how participants who did not use the application or dropped out from the trial were treated in the statistical analysis (a complete case analysis is strongly discouraged, and simple imputation techniques such as LOCF may also be problematic [4]).

	1	2	3	4	5	
subitem not at all important	○	○	○	○	◉	essential

Does your paper address subitem 12a-i? *

Copy and paste relevant sections from the manuscript (include quotes in quotation marks "like this" to indicate direct quotes from your manuscript), or elaborate on this item by providing additional information not in the ms, or briefly explain why the item is not applicable/relevant for your study

"The approach to the outcome analysis was Intention-to-Treat. First, we analyzed the data without any imputation for missing values and then repeated the analysis with an imputation of missing values using a last observation carried forward (LOCF) method. The results were similar for the complete-case analysis and analysis with LOCF; both are reported (see Multimedia Appendices 1 and 2)."

"Scales were calculated as follows: if the number of missing items was more than half of the number of items of a scale, the scale was considered missing; otherwise the missing items were imputed in accord with the average of the nonmissing items of the scale."

12b) Methods for additional analyses, such as subgroup analyses and adjusted analyses

Does your paper address CONSORT subitem 12b? *

Copy and paste relevant sections from the manuscript (include quotes in quotation marks "like this" to indicate direct quotes from your manuscript), or elaborate on this item by providing additional information not in the ms, or briefly explain why the item is not applicable/relevant for your study

We could not conduct sub-analysis, such as by gender, given modest sample size. This is acknowledged in the discussion by recommending future studies with larger sample and from multiple sites.

X26) REB/IRB Approval and Ethical Considerations [recommended as subheading under "Methods"] (not a CONSORT item)

X26-i) Comment on ethics committee approval

	1	2	3	4	5	
subitem not at all important	○	○	○	○	○	essential

Does your paper address subitem X26-i?

Copy and paste relevant sections from the manuscript (include quotes in quotation marks "like this" to indicate direct quotes from your manuscript), or elaborate on this item by providing additional information not in the ms, or briefly explain why the item is not applicable/relevant for your study

Your answer

x26-ii) Outline informed consent procedures

Outline informed consent procedures e.g., if consent was obtained offline or online (how? Checkbox, etc.?), and what information was provided (see 4a-ii). See [6] for some items to be included in informed consent documents.

	1	2	3	4	5	
subitem not at all important	○	○	○	○	○	essential

Does your paper address subitem X26-ii?

Copy and paste relevant sections from the manuscript (include quotes in quotation marks "like this" to indicate direct quotes from your manuscript), or elaborate on this item by providing additional information not in the ms, or briefly explain why the item is not applicable/relevant for your study

Your answer

X26-iii) Safety and security procedures

Safety and security procedures, incl. privacy considerations, and any steps taken to reduce the likelihood or detection of harm (e.g., education and training, availability of a hotline)

	1	2	3	4	5	
subitem not at all important	○	○	○	○	○	essential

Does your paper address subitem X26-iii?

Copy and paste relevant sections from the manuscript (include quotes in quotation marks "like this" to indicate direct quotes from your manuscript), or elaborate on this item by providing additional information not in the ms, or briefly explain why the item is not applicable/relevant for your study

Your answer

RESULTS

13a) For each group, the numbers of participants who were randomly assigned, received intended treatment, and were analysed for the primary outcome

NPT: The number of care providers or centers performing the intervention in each group and the number of patients treated by each care provider in each center

Does your paper address CONSORT subitem 13a? *

Copy and paste relevant sections from the manuscript (include quotes in quotation marks "like this" to indicate direct quotes from your manuscript), or elaborate on this item by providing additional information not in the ms, or briefly explain why the item is not applicable/relevant for your study

Yes, please see figure 3, Study Flow Diagram. Result section states "A total of 119 undergraduate students were randomized to the WLC, F-MVC, and P-MVC groups; 1 participant, following consent, was found to be underage and was therefore excluded, and 5 additional participants were nonrespondents to the T1 survey. Out of the 113 students who completed the T1 survey, a few were lost as nonrespondents at the follow-up; altogether the attrition was relatively low across all three groups (F-MVC: 2/39, P-MVC: 1/35, and WLC: 1/39; Figure 3)."

13b) For each group, losses and exclusions after randomisation, together with reasons

Does your paper address CONSORT subitem 13b? (NOTE: Preferably, this is shown in a CONSORT flow diagram) *

Copy and paste relevant sections from the manuscript (include quotes in quotation marks "like this" to indicate direct quotes from your manuscript), or elaborate on this item by providing additional information not in the ms, or briefly explain why the item is not applicable/relevant for your study

Result section states "A total of 119 undergraduate students were randomized to the WLC, F-MVC, and P-MVC groups; 1 participant, following consent, was found to be underage and was therefore excluded, and 5 additional participants were nonrespondents to the T1 survey. Out of the 113 students who completed the T1 survey, a few were lost as nonrespondents at the follow-up; altogether the attrition was relatively low across all three groups (F-MVC: 2/39, P-MVC: 1/35, and WLC:

13b-i) Attrition diagram

Strongly recommended: An attrition diagram (e.g., proportion of participants still logging in or using the intervention/comparator in each group plotted over time, similar to a survival curve) or other figures or tables demonstrating usage/dose/engagement.

	1	2	3	4	5	
subitem not at all important	○	○	○	○	○	essential

Does your paper address subitem 13b-i?

Copy and paste relevant sections from the manuscript or cite the figure number if applicable (include quotes in quotation marks "like this" to indicate direct quotes from your manuscript), or elaborate on this item by providing additional information not in the ms, or briefly explain why the item is not applicable/relevant for your study

Your answer

14a) Dates defining the periods of recruitment and follow-up

Does your paper address CONSORT subitem 14a? *

Copy and paste relevant sections from the manuscript (include quotes in quotation marks "like this" to indicate direct quotes from your manuscript), or elaborate on this item by providing additional information not in the ms, or briefly explain why the item is not applicable/relevant for your study

“The recruitment of eligible undergraduate students occurred during December 7, 2016 and January 10, 2017. These students started the parallel-arm RCT on January 16, 2017, with a baseline survey (T1) followed by exposure to 2 interventions, a 4-week online survey (T2), and an 8-week online survey (T3). The 8-week-long interventions of F-MVC and P-MVC started on January 22, 2017 and

14a-i) Indicate if critical “secular events” fell into the study period

Indicate if critical “secular events” fell into the study period, e.g., significant changes in Internet resources available or “changes in computer hardware or Internet delivery resources”

	1	2	3	4	5	
subitem not at all important	○	○	○	○	○	essential

Does your paper address subitem 14a-i?

Copy and paste relevant sections from the manuscript (include quotes in quotation marks "like this" to indicate direct quotes from your manuscript), or elaborate on this item by providing additional information not in the ms, or briefly explain why the item is not applicable/relevant for your study

Your answer

14b) Why the trial ended or was stopped (early)

Does your paper address CONSORT subitem 14b? *

Copy and paste relevant sections from the manuscript (include quotes in quotation marks "like this" to indicate direct quotes from your manuscript), or elaborate on this item by providing additional information not in the ms, or briefly explain why the item is not applicable/relevant for your study

Not applicable

15) A table showing baseline demographic and clinical characteristics for each group

NPT: When applicable, a description of care providers (case volume, qualification, expertise, etc.) and centers (volume) in each group

Does your paper address CONSORT subitem 15? *

Copy and paste relevant sections from the manuscript (include quotes in quotation marks "like this" to indicate direct quotes from your manuscript), or elaborate on this item by providing additional information not in the ms, or briefly explain why the item is not applicable/relevant for your study

See following tables:
Table 2. Participant characteristics.
Table 3. Symptom levels for depression, anxiety, and stress scales.
Figure 4. Box plots for the mean scores of depression, anxiety, and stress scales.
Table 4. Mean (SD) and effect size for depression, anxiety, and stress scales.
Table 5. Mean (SD) and effect size for quality of life, life satisfaction, and mindfulness scales.

15-i) Report demographics associated with digital divide issues

In ehealth trials it is particularly important to report demographics associated with digital divide issues, such as age, education, gender, social-economic status, computer/Internet/ehealth literacy of the participants, if known.

	1	2	3	4	5	
subitem not at all important	○	○	○	○	○	essential

Does your paper address subitem 15-i? *

Copy and paste relevant sections from the manuscript (include quotes in quotation marks "like this" to indicate direct quotes from your manuscript), or elaborate on this item by providing additional information not in the ms, or briefly explain why the item is not applicable/relevant for your study

We could not do such analysis given modest sample size. Further, all participants were university students so their digital skills are likely to be quite similar.

16) For each group, number of participants (denominator) included in each analysis and whether the analysis was by original assigned groups

16-i) Report multiple "denominators" and provide definitions

Report multiple "denominators" and provide definitions: Report N's (and effect sizes) "across a range of study participation [and use] thresholds" [1], e.g., N exposed, N consented, N used more than x times, N used more than y weeks, N participants "used" the intervention/comparator at specific pre-defined time points of interest (in absolute and relative numbers per group). Always clearly define "use" of the intervention.

	1	2	3	4	5	
subitem not at all important	○	○	○	○	○	essential

Does your paper address subitem 16-i? *

Copy and paste relevant sections from the manuscript (include quotes in quotation marks "like this" to indicate direct quotes from your manuscript), or elaborate on this item by providing additional information not in the ms, or briefly explain why the item is not applicable/relevant for your study

The tables provide "N" for each group in the trial and the Study Flow Diagram shows the participants, approached, randomized, lost and analyzed.

16-ii) Primary analysis should be intent-to-treat

Primary analysis should be intent-to-treat, secondary analyses could include comparing only “users”, with the appropriate caveats that this is no longer a randomized sample (see 18-i).

	1	2	3	4	5	
subitem not at all important	○	○	○	○	○	essential

Does your paper address subitem 16-ii?

Copy and paste relevant sections from the manuscript (include quotes in quotation marks "like this" to indicate direct quotes from your manuscript), or elaborate on this item by providing additional information not in the ms, or briefly explain why the item is not applicable/relevant for your study

Your answer

17a) For each primary and secondary outcome, results for each group, and the estimated effect size and its precision (such as 95% confidence interval)

Does your paper address CONSORT subitem 17a? *

Copy and paste relevant sections from the manuscript (include quotes in quotation marks "like this" to indicate direct quotes from your manuscript), or elaborate on this item by providing additional information not in the ms, or briefly explain why the item is not applicable/relevant for your study

We provide 95% CI for table 3, effect sizes in table 4 and 5, and SE for table 6 and 7 for adjusted and unadjusted GEE.

17a-i) Presentation of process outcomes such as metrics of use and intensity of use

In addition to primary/secondary (clinical) outcomes, the presentation of process outcomes such as metrics of use and intensity of use (dose, exposure) and their operational definitions is critical. This does not only refer to metrics of attrition (13-b) (often a binary variable), but also to more continuous exposure metrics such as “average session length”. These must be accompanied by a technical description how a metric like a “session” is defined (e.g., timeout after idle time) [1] (report under item 6a).

	1	2	3	4	5	
subitem not at all important	○	○	○	○	○	essential

Does your paper address subitem 17a-i?

Copy and paste relevant sections from the manuscript (include quotes in quotation marks "like this" to indicate direct quotes from your manuscript), or elaborate on this item by providing additional information not in the ms, or briefly explain why the item is not applicable/relevant for your study

Your answer

17b) For binary outcomes, presentation of both absolute and relative effect sizes is recommended

Does your paper address CONSORT subitem 17b? *

Copy and paste relevant sections from the manuscript (include quotes in quotation marks "like this" to indicate direct quotes from your manuscript), or elaborate on this item by providing additional information not in the ms, or briefly explain why the item is not applicable/relevant for your study

The primary and secondary outcomes are analyzed using their mean scores and the change in mean scores.

18) Results of any other analyses performed, including subgroup analyses and adjusted analyses, distinguishing pre-specified from exploratory

Does your paper address CONSORT subitem 18? *

Copy and paste relevant sections from the manuscript (include quotes in quotation marks "like this" to indicate direct quotes from your manuscript), or elaborate on this item by providing additional information not in the ms, or briefly explain why the item is not applicable/relevant for your study

All primary (depression, anxiety, stress) and secondary outcomes (life satisfaction, quality of life, mindfulness and academic performance/class absenteeism) were registered before data collection.

18-i) Subgroup analysis of comparing only users

A subgroup analysis of comparing only users is not uncommon in ehealth trials, but if done, it must be stressed that this is a self-selected sample and no longer an unbiased sample from a randomized trial (see 16-iii).

	1	2	3	4	5	
subitem not at all important	○	○	○	○	○	essential

Does your paper address subitem 18-i?

Copy and paste relevant sections from the manuscript (include quotes in quotation marks "like this" to indicate direct quotes from your manuscript), or elaborate on this item by providing additional information not in the ms, or briefly explain why the item is not applicable/relevant for your study

Your answer

19) All important harms or unintended effects in each group

(for specific guidance see CONSORT for harms)

Does your paper address CONSORT subitem 19? *

Copy and paste relevant sections from the manuscript (include quotes in quotation marks "like this" to indicate direct quotes from your manuscript), or elaborate on this item by providing additional information not in the ms, or briefly explain why the item is not applicable/relevant for your study

"Although our study participants largely comprised healthy volunteers, our protocol included a safety mechanism whereby participants were asked verbally and in the consent form to contact the research staff if they felt distress during the trial period so that "limited counselling with a clinical psychologist could be arranged, if needed"; the collaborating psychologist was at arms' length from the trial. No instance of such request arose during the reported study."

There were no harms or unintended effects reported by the participants during the study period.

19-i) Include privacy breaches, technical problems

Include privacy breaches, technical problems. This does not only include physical “harm” to participants, but also incidents such as perceived or real privacy breaches [1], technical problems, and other unexpected/unintended incidents. “Unintended effects” also includes unintended positive effects [2].

	1	2	3	4	5	
subitem not at all important	○	○	○	○	○	essential

Does your paper address subitem 19-i?

Copy and paste relevant sections from the manuscript (include quotes in quotation marks "like this" to indicate direct quotes from your manuscript), or elaborate on this item by providing additional information not in the ms, or briefly explain why the item is not applicable/relevant for your study

Your answer

19-ii) Include qualitative feedback from participants or observations from staff/researchers

Include qualitative feedback from participants or observations from staff/researchers, if available, on strengths and shortcomings of the application, especially if they point to unintended/unexpected effects or uses. This includes (if available) reasons for why people did or did not use the application as intended by the developers.

	1	2	3	4	5	
subitem not at all important	○	○	○	○	○	essential

Does your paper address subitem 19-ii?

Copy and paste relevant sections from the manuscript (include quotes in quotation marks "like this" to indicate direct quotes from your manuscript), or elaborate on this item by providing additional information not in the ms, or briefly explain why the item is not applicable/relevant for your study

Your answer

DISCUSSION

22) Interpretation consistent with results, balancing benefits and harms, and considering other relevant evidence

NPT: In addition, take into account the choice of the comparator, lack of or partial blinding, and unequal expertise of care providers or centers in each group

22-i) Restate study questions and summarize the answers suggested by the data, starting with primary outcomes and process outcomes (use)

Restate study questions and summarize the answers suggested by the data, starting with primary outcomes and process outcomes (use).

	1	2	3	4	5	
subitem not at all important	○	○	○	○	○	essential

Does your paper address subitem 22-i? *

Copy and paste relevant sections from the manuscript (include quotes in quotation marks "like this" to indicate direct quotes from your manuscript), or elaborate on this item by providing additional information not in the ms, or briefly explain why the item is not applicable/relevant for your study

Yes, see section on "Principal Findings" under the discussion.

22-ii) Highlight unanswered new questions, suggest future research

Highlight unanswered new questions, suggest future research.

	1	2	3	4	5	
subitem not at all important	○	○	○	○	○	essential

Does your paper address subitem 22-ii?

Copy and paste relevant sections from the manuscript (include quotes in quotation marks "like this" to indicate direct quotes from your manuscript), or elaborate on this item by providing additional information not in the ms, or briefly explain why the item is not applicable/relevant for your study

Your answer

20) Trial limitations, addressing sources of potential bias, imprecision, and, if relevant, multiplicity of analyses

20-i) Typical limitations in ehealth trials

Typical limitations in ehealth trials: Participants in ehealth trials are rarely blinded. Ehealth trials often look at a multiplicity of outcomes, increasing risk for a Type I error. Discuss biases due to non-use of the intervention/usability issues, biases through informed consent procedures, unexpected events.

	1	2	3	4	5	
subitem not at all important	○	○	○	○	○	essential

Does your paper address subitem 20-i? *

Copy and paste relevant sections from the manuscript (include quotes in quotation marks "like this" to indicate direct quotes from your manuscript), or elaborate on this item by providing additional information not in the ms, or briefly explain why the item is not applicable/relevant for your study

Yes, see "Strengths and Limitations" under the discussion.

21) Generalisability (external validity, applicability) of the trial findings

NPT: External validity of the trial findings according to the intervention, comparators, patients, and care providers or centers involved in the trial

21-i) Generalizability to other populations

Generalizability to other populations: In particular, discuss generalizability to a general Internet population, outside of a RCT setting, and general patient population, including applicability of the study results for other organizations

	1	2	3	4	5	
subitem not at all important	○	○	○	○	○	essential

Does your paper address subitem 21-i?

Copy and paste relevant sections from the manuscript (include quotes in quotation marks "like this" to indicate direct quotes from your manuscript), or elaborate on this item by providing additional information not in the ms, or briefly explain why the item is not applicable/relevant for your study

Your answer

21-ii) Discuss if there were elements in the RCT that would be different in a routine application setting

Discuss if there were elements in the RCT that would be different in a routine application setting (e.g., prompts/reminders, more human involvement, training sessions or other co-interventions) and what impact the omission of these elements could have on use, adoption, or outcomes if the intervention is applied outside of a RCT setting.

	1	2	3	4	5	
subitem not at all important	○	○	○	○	○	essential

Does your paper address subitem 21-ii?

Copy and paste relevant sections from the manuscript (include quotes in quotation marks "like this" to indicate direct quotes from your manuscript), or elaborate on this item by providing additional information not in the ms, or briefly explain why the item is not applicable/relevant for your study

Your answer

OTHER INFORMATION

23) Registration number and name of trial registry

Does your paper address CONSORT subitem 23? *

Copy and paste relevant sections from the manuscript (include quotes in quotation marks "like this" to indicate direct quotes from your manuscript), or elaborate on this item by providing additional information not in the ms, or briefly explain why the item is not applicable/relevant for your study

"Trial Registration: International Standard Randomized Controlled Trial Number ISRCTN92827275; https://www.isrctn.com/ISRCTN92827275"

24) Where the full trial protocol can be accessed, if available

Does your paper address CONSORT subitem 24? *

Cite a Multimedia Appendix, other reference, or copy and paste relevant sections from the manuscript (include quotes in quotation marks "like this" to indicate direct quotes from your manuscript), or elaborate on this item by providing additional information not in the ms, or briefly explain why the item is not applicable/relevant for your study

"Trial Registration: International Standard Randomized Controlled Trial Number ISRCTN92827275; https://www.isrctn.com/ISRCTN92827275"

25) Sources of funding and other support (such as supply of drugs), role of funders

Does your paper address CONSORT subitem 25? *

Copy and paste relevant sections from the manuscript (include quotes in quotation marks "like this" to indicate direct quotes from your manuscript), or elaborate on this item by providing additional information not in the ms, or briefly explain why the item is not applicable/relevant for your study

"Acknowledgments
The authors acknowledge the contribution of all the students who gave their valuable time to participate in the study. They would like to thank ForaHealthyme Inc as an industry partner in this project.

The work reported in this paper was funded by the Canadian Institutes for Health Research (CIHR), eHealth Innovations Partnership Program Grant; grant number EH1-143553. The project principal investigators are CE (nominated), FA, and PR. FA was partially supported by the CIHR New Investigator Award. The Early Researcher Award from the Ministry of Economic Development, Job Creation and Trade (held by FA) supported NO as postdoctoral fellow."

X27) Conflicts of Interest (not a CONSORT item)

X27-i) State the relation of the study team towards the system being evaluated

In addition to the usual declaration of interests (financial or otherwise), also state the relation of the study team towards the system being evaluated, i.e., state if the authors/evaluators are distinct from or identical with the developers/sponsors of the intervention.

	1	2	3	4	5	
subitem not at all important	○	○	○	○	◉	essential

Does your paper address subitem X27-i?

Copy and paste relevant sections from the manuscript (include quotes in quotation marks "like this" to indicate direct quotes from your manuscript), or elaborate on this item by providing additional information not in the ms, or briefly explain why the item is not applicable/relevant for your study

"Conflicts of Interest
It is the understanding of the university and researchers that the Project Intellectual Property belongs to the CE, FA, and PR. The industry partner ForaHealthyme has title to the copyrights of any computer source code software that was developed out of this

About the CONSORT EHEALTH checklist

As a result of using this checklist, did you make changes in your manuscript? *

- ○ yes, major changes
- ● yes, minor changes
- ○ no

What were the most important changes you made as a result of using this checklist?

description of intervention development and its components.

How much time did you spend on going through the checklist INCLUDING making changes in your manuscript *

5 hours

As a result of using this checklist, do you think your manuscript has improved? *

- ● yes
- ○ no
- ○ Other:

Would you like to become involved in the CONSORT EHEALTH group?

This would involve for example becoming involved in participating in a workshop and writing an "Explanation and Elaboration" document

- ( ) yes
- (x) no
- ( ) Other:

Any other comments or questions on CONSORT EHEALTH

Please split the form into modules. There are sometimes Internet connections that could lead to loss of completed sections of the form.

STOP - Save this form as PDF before you click submit

To generate a record that you filled in this form, we recommend to generate a PDF of this page (on a Mac, simply select "print" and then select "print as PDF") before you submit it.

When you submit your (revised) paper to JMIR, please upload the PDF as supplementary file.

Don't worry if some text in the textboxes is cut off, as we still have the complete information in our database. Thank you!

Final step: Click submit !

Click submit so we have your answers in our database!

Submit

Never submit passwords through Google Forms.

This content is neither created nor endorsed by Google. Report Abuse - Terms of Service - Privacy Policy

Google Forms
